# Potato root-associated microbiomes adapt to combined water and nutrient limitation and have a plant genotype-specific role for plant stress mitigation

**DOI:** 10.1186/s40793-023-00469-x

**Published:** 2023-03-14

**Authors:** Hanna Faist, Friederike Trognitz, Livio Antonielli, Sarah Symanczik, Philip J. White, Angela Sessitsch

**Affiliations:** 1 Bioresources Unit, AIT Austrian Institute of Technology, Konrad-Lorenz-Straße 24, 3430 Tulln, Austria; 2 Soil Science Department, Research Institute of Organic Agriculture (FiBL), Ackerstraße 113, 5070 Frick, Switzerland; 3 The James Hutton Institute, Invergowrie, Dundee, DD2 5DA UK

**Keywords:** Shotgun metagenomics, *Solanum tuberosum*, *Solanum phureja*, Bacteriophage, Plant–microbe interaction, Plasmid, Rhizosphere, Endophytes, Rhizobacteria, Drought

## Abstract

**Background:**

Due to climate change and reduced use of fertilizers combined stress scenarios are becoming increasingly frequent in crop production. In a field experiment we tested the effect of combined water and phosphorus limitation on the growth performance and plant traits of eight tetraploid and two diploid potato varieties as well as on root-associated microbiome diversity and functional potential. Microbiome and metagenome analysis targeted the diversity and potential functions of prokaryotes, fungi, plasmids, and bacteriophages and was linked to plant traits like tuber yield or timing of canopy closure.

**Results:**

The different potato genotypes responded differently to the combined stress and hosted distinct microbiota in the rhizosphere and the root endosphere. Proximity to the root, stress and potato genotype had significant effects on bacteria, whereas fungi were only mildly affected. To address the involvement of microbial functions, we investigated well and poorly performing potato genotypes (Stirling and Desirée, respectively) under stress conditions and executed a metagenome analysis of rhizosphere microbiomes subjected to stress and no stress conditions. Functions like ROS detoxification, aromatic amino acid and terpene metabolism were enriched and in synchrony with the metabolism of stressed plants. In Desirée, *Pseudonocardiale*s had the genetic potential to take up assimilates produced in the fast-growing canopy and to reduce plant stress-sensing by degrading ethylene, but overall yield losses were high. In Stirling, *Xanthomonadale*s had the genetic potential to reduce oxidative stress and to produce biofilms, potentially around roots. Biofilm formation could be involved in drought resilience and nutrient accessibility of Stirling and explain the recorded low yield losses. In the rhizosphere exposed to combined stress, the relative abundance of plasmids was reduced, and the diversity of phages was enriched. Moreover, mobile elements like plasmids and phages were affected by combined stresses in a genotype-specific manner.

**Conclusion:**

Our study gives new insights into the interconnectedness of root-associated microbiota and plant stress responses in the field. Functional genes in the metagenome, phylogenetic composition and mobile elements play a role in potato stress adaption. In a poor and a well performing potato genotype grown under stress conditions, distinct functional genes pinpoint to a distinct stress sensing, water availability and compounds in the rhizospheres.

**Supplementary Information:**

The online version contains supplementary material available at 10.1186/s40793-023-00469-x.

## Background

Potato (*Solanum tuberosum*) is the world´s 4th most produced staple crop, after maize, wheat, and rice. Potato production has been considered to be severely impacted due to global warming and drought and a yield decline of 18–32% in the period between 2040 and 2069 has been predicted [1]. Also, this crop typically has a high phosphorus (P) demand and low P uptake efficiency [2], and P is required for early plant development and for tuber production [3]. Efforts are ongoing to improve tolerance of potato to various abiotic stresses, primarily to drought, by intensive crop breeding, e.g., by enhancing photosynthetic performance [4]. In addition, the plant microbiome has the potential to alleviate plant stress [5] and may be modulated to improve potato production.

Plant microbiomes, i.e., plant-associated microbial communities and their “theatre of activity” [6] are highly complex, consisting of bacteria, archaea, fungi, oomycetes, protists, and viruses, and can be essentially found in all plant tissues and compartments. Plant microbiota are important for plant growth and health and are involved in key functions such as nutrient mobilization, protection against pathogens or improving plant resilience to abiotic stress [7]. Well studied plant compartments for microbial life include the rhizosphere [8] the endosphere [9] and the phyllosphere [10]. The rhizosphere is a hotspot of microbial diversity and activity utilizing root exudates and sloughed off plant cells as nutrient sources [8]. After colonizing the rhizoplane microorganisms may also enter roots, either passively e.g., via wounds or actively using cellulolytic enzymes and may thrive in the root endosphere and/or translocate to above-ground plant tissues [9]. Microbial communities in different plant compartments are clearly different from each other, indicating strong influence of host-specific factors [7, 9]. Whereas microbiota components of the rhizosphere at a coarse level are similar for various plant species, there is generally a greater influence of the host plant on endophytes [7]. In addition, stress conditions, plant development and the plant genotype are major drivers of plant microbiota [11, 12].

Recent work has indicated that also soil microbiomes alter plant fitness and competition under drought [13] and that root microbiome compositional changes correlate with drought stress tolerance across plant species [14]. Several studies have shown that drought affects the composition of root microbiota, particularly favouring monoderm bacteria such as Actinobacteria, which are known to be more resistant to desiccation than diderms [13–16]. The effect of plant phosphorus starvation on plant microbiomes is less well investigated. Finkel et al. [17] recently showed that the phosphate starvation response of *Arabidopsis* has a large effect on the plant-associated bacterial and fungal communities, whereas different types of P-fertilizers did not show a major influence on below-ground microbial communities [18].

Only few microbial mechanisms are known to be responsible for alleviating plant drought stress. Bacteria producing the enzyme ACC deaminase are prominent candidates for the improvement of drought stress resilience [19]. This enzyme is responsible for lowering the levels of ethylene in the plant by cleaving the plant-produced ethylene precursor 1-aminocyclopropane-1-carboxylate (ACC) to ammonia and 2-oxobutanoate, modulating ethylene signalling [20]. Other known mechanisms include the detoxification of reactive oxygen species (ROS) [21, 22] or modulating abscisic acid metabolism [23]. With regard to supporting plants in P acquisition, microorganisms are known to solubilize poorly available P pools. Particularly phosphatases released by microorganisms mediate mobilization of soil P via mineralization of organic P [24].

The plant genotype greatly determines plant traits like resilience to drought [25] or P utilization efficiency [3], but also influences microbiome structure and functions [26, 27]. Very recently, it has been proposed to rather focus on the host phenotype rather than genotype as a predictor and readout of microbiome function [28]. This proposed view also supports the underlying hypothesis of our work, i.e., that—together with other parameters such as stress—the potato genotype and particularly the phenotype direct the structure and functions of the associated root and rhizosphere microbiome. Along these lines we assessed various phenotypic traits of ten field-grown *Solanum tuberosum* (eight tetraploids belonging to group Tuberosum; two belonging to the diploid group Phureja) genotypes, particularly those related to the resilience to combined water stress and P limitation and related these traits to the plant microbiome. We furthermore had the hypothesis that plant genotypes showing a contrasting phenotype (like stress resilience) host distinct microbiota equipped with different functions to interact with plants and to support their stress resilience. To address this, we performed a metagenomic analysis of rhizosphere microbiomes of two contrasting genotypes and particularly investigated potential functions involved in the observed plant phenotype. As plasmids and phages have been reported to act as drivers of ecological and evolutionary processes [29, 30] and are important mediators of horizontal gene transfer, we also used the metagenomic data to elaborate the effects of stress and plant genotype on plasmids and phage communities.

## Results

Ten potato genotypes (Additional file 1: Table S1) were grown in the field with a combined stress of reduced irrigation and no phosphate fertilizer (Additional file 1: Fig. S1A, B), termed here “combined stress” for simplicity. For all measured time points, soil moisture was highest in the deep soil layers and reduced in the top soil and was different between stress conditions (Additional file 1: Fig. S1C, D). Comparing the number of young tubers eight weeks after planting, half time to canopy closure, final yield, and above ground biomass (foliage) of potato grown under combined stress and under conditions without water and P limitation revealed that different genotypes exhibited different stress responses. The stress effect on tuber yield correlated significantly with the effect on above-ground biomass (Additional file 1: Fig. SlE) but a reduced number of young tubers during tuber filling correlated with stress resilience in tuber yield (Additional file 1: Fig. SlF). This indicates that a delay in growth under continuous but reduced water supply and P limitation is beneficial for stress resilience of the potato plants.

High throughput amplicon sequencing of 166 samples revealed a total of 5.8 M 16S rRNA gene sequences and 3.6 M ITS sequences after removing plant-derived sequences. Those were grouped into 20,114 (1302 occurring in at least three samples) different bacterial and 941 (76) fungal amplicon sequence variants (ASVs). On average the samples contained 34 k ± 19 k bacterial and 21 k ± 18 k fungal ASVs. From two contrasting genotypes (Desirée = high yield loss under combined stress, Stirling = low yield loss under combined stress), we analysed the functional potential of the rhizosphere microbial communities of plants grown under the different stress conditions by shotgun metagenomics. We obtained 136 M ± 17 M sequences per sample and in total 1633 M paired sequences. A 98% subset of all classified reads belonged to bacteria, 1.2% to phages, 0.7% to archaea and 0.1% to fungi. Reads of plasmids summed up to 6.2% of all reads classified with the Kraken-Braken method.

### Sample type and stress shape the microbial diversity and structure of potatoes

**Fig. 1:**
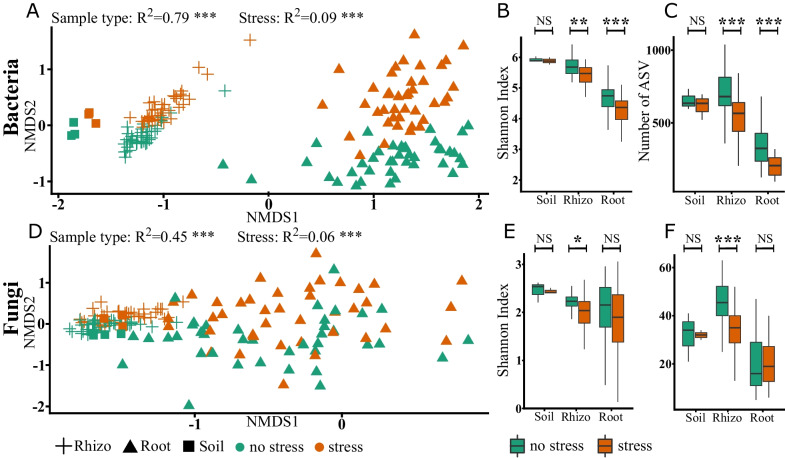
Microbial diversity of potato plants. Beta-diversity of the 166 potato-associated microbiota is shown in a non-metric multidimensional scaling (NMDS) ordination based on the Bray–Curtis distance according to sample type (shape of the symbols) and stress (colour) (**A**, **D**). Samples with similar composition cluster. Significance was calculated using a permutation test. Alpha-diversity indicated (**B**, **E**) by the Shannon index and (**C**, **F**) the number of different ASVs, the richness of a sample. Significance was determined by Wilcoxon-tests. * <  = 0.1, ** <  = 0.05, *** <  = 0.01 NS = not significant

The nonmetric multidimensional scaling (NMDS) ordination of the amplicon dataset showed that the structural differences in the microbial community composition were mostly influenced by the sample type (Fig. 1A, D environmental fit, bacteria: R2 = 0.79, *p* value ≤ 0.001, fungi: R2 = 0.45, *p* value ≤ 0.001) followed by stress (bacteria: R2 = 0.09, *p* value ≤ 0.001, fungi: R2 = 0.06, *p* value ≤ 0.001). The calculation of a general linear model of the values of NMDS1 resulted in a significant influence of each sample type on the bacterial composition (soil, *p *value < 0.001; rhizosphere, *p *value < 0.001; root, *p *value < 0.001) but the fungal rhizosphere composition did not differ significantly from the other sample types (soil, *p *value < 0.001; rhizosphere, *p *value = 0.94; root, *p *value < 0.001). A general linear model of the scores of NMDS2 suggested a significant influence of combined stress on the microbiota (bacteria: stress, *p *value < 0.001; no stress, *p *value < 0.001; fungi: stress, *p *value < 0.001; no stress, *p *value < 0.001). The highest richness (= number of different amplicon sequences) was found in the rhizosphere followed by soil and root samples (Fig. 1C, F). Both, richness and Shannon Index, revealed a reduction in microbial diversity in the rhizosphere under combined stress conditions. In roots a significant reduction was only observed for bacteria (Fig. 1B, E). Additionally, the diversity of archaea, which were analysed by metagenomics only, was increased under combined stress (Shannon Index: stress = 3.47, no stress = 3.27, *p *value = 0.015).

### Common stress reactions of the microbial composition in various potato genotypes

**Fig. 2:**
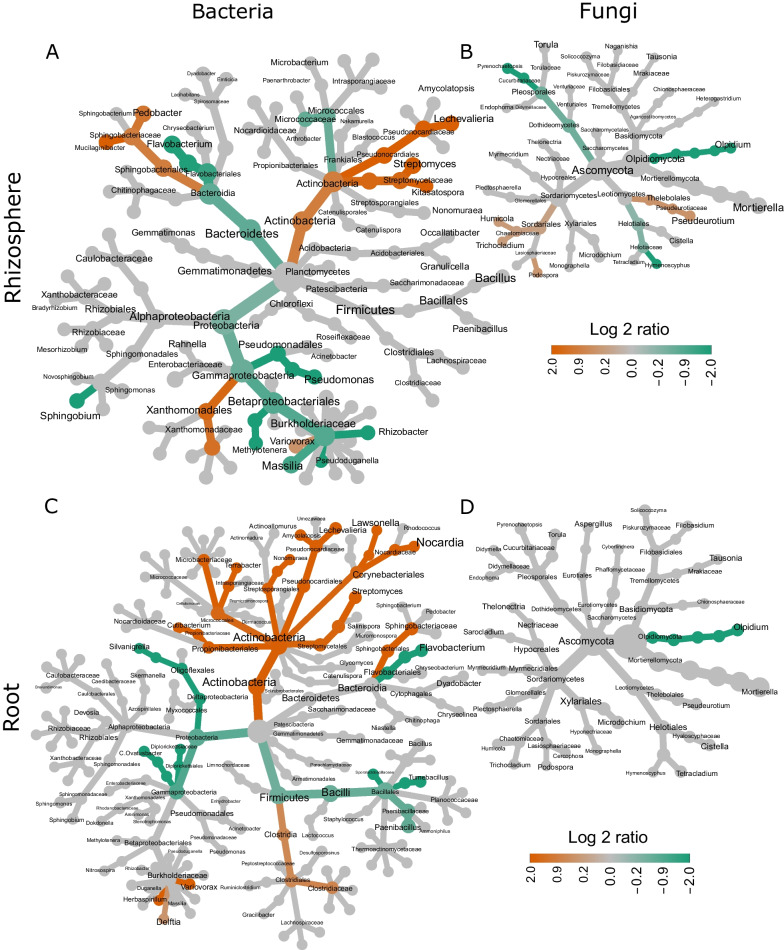
Changes in microbial composition according to stress. Fungal (**B**, **D**) and bacterial **A**, **C**) composition of rhizosphere (**A**, **B**) and root (**C**, **D**) samples. Each node represents a taxonomic rank. Different taxonomic ranks are shown, starting from the highest rank (largest grey nodes) to the genus level at the end of the branches. Coloured nodes represent taxa which are significantly enriched (orange) or reduced (green) under stress (Wilcoxon test <  = 0.05)

The most abundant phyla in the rhizosphere included Proteobacteria, Actinobacteria, Bacteroidetes, Ascomycota, Mortierellomycota and Basidiomycota (Additional file 1: Fig. S2). At the genus level the rhizosphere contained mostly *Sphingomonas*, *Flavobacterium, Streptomyces, Mortiella, Solicoccozyma* and *Pseudeurotium*. Roots were additionally dominated by the phyla Firmicutes as well as Olpidiomycota and by the genera *Bacillus, Paenibacillus* and *Microdochium* (Additional file 1: Fig. S3). Under combined stress conditions Actinobacteria, Sphingobacteriales and *Variovorax* were enriched, while Proteobacteria, Flavobacteriales and Olpidiomycota were reduced in roots and the rhizosphere. We observed sample type-specific stress reactions like the enrichment of Xanthomonadales in rhizosphere samples and Clostridia in roots under stress conditions. In contrast to Xanthomonadales and Clostridia, the abundance of other Gammaproteobacteria and Firmicutes was reduced under combined stress (Fig. 2). Similarly, different members of the Leotiomycetes showed different responses, Theloboales were enriched and Helotiales were reduced in the rhizosphere under combined stress.

**Fig. 3:**
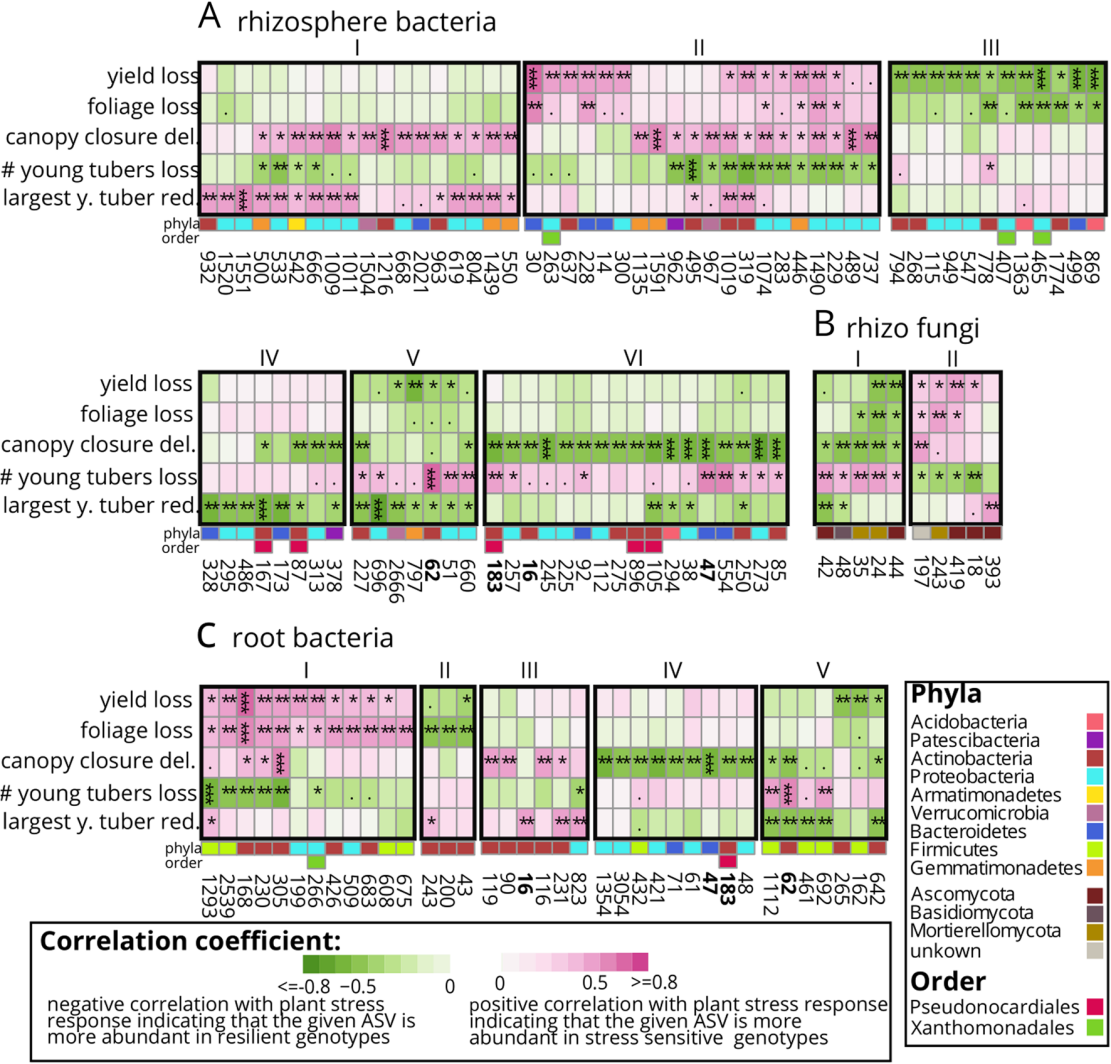
Key microbiota (**A** rhizosphere bacteria; **B** rhizosphere fungi; **C** root bacteria) in stress resilient and suffering genotypes. Columns represent microbes, which are numbered according to their amplicon sequencing variant (ASV) and taxonomical position classified in Additional file 1: Table S8. Rows indicate the strength of potato plant stress responses. This includes percentual tuber yield loss, weight of foliage loss, delay (del.) of half-time canopy closure, differences in number (#) of young tubers and differences in the diameter of the largest young (y.) tuber. Dark pink indicates a high abundance of a specific ASV under stress in potato plants suffering in this phenotype, while green indicates a high abundance in potato plants resilient in this phenotype. Black frames with Roman numbers, indicate clusters of ASVs correlating with the same stress response pattern. Bold ASV-numbers refer to ASVs that correlate with a stress response in roots and rhizosphere samples. Significant spearman correlations are determined by a t-test and results are indicated: *p *value < 0.001: ***; *p *val < 0.01: **; *p *val < 0.05: *, *p *val < 0.1

At the highest taxonomic resolution of the amplicon-dataset, we identified 174 ASVs showing significantly different abundance in one of the two stress treatments (Additional file 1: Table S2): (i) root endosphere: 6 fungal and 46 bacterial ASVs; (ii) rhizosphere: 4 fungal and 118 bacterial ASVs. Interestingly only three ASVs, all belonging to Actinobacteria, were significantly enriched in both root and rhizosphere samples under combined stress: *Nonomuraea* sp. ASV_90, *Streptomyces* sp. ASV_268 and *Streptomyces* sp. ASV_9.

The reduced shotgun-dataset confirmed the enrichment of Actinobacteria and other stress-specific bacterial taxa but detected more significant differences in Alphaproteobacteria compared to the amplicon dataset (Additional file 1: Fig. S4A). Regarding archaea in the reduced shotgun-dataset, Methanococci were more abundant under cultivation conditions without water and P limitation, while Halobacteriales, Haloferacales and Methanomicrobia were enriched under combined stress (Additional file 1: Fig. S4B). In total 17 good quality metagenome assembled genomes (MAGs) were identified (Additional file 1: Table S3). Five MAGs (3 Actinobacteria, 2 Proteobacteria) were more abundant and four (all Proteobacteria, genus *Sphingobium*) were depleted under combined stress (Additional file 1: Table S4).

**Fig. 4:**
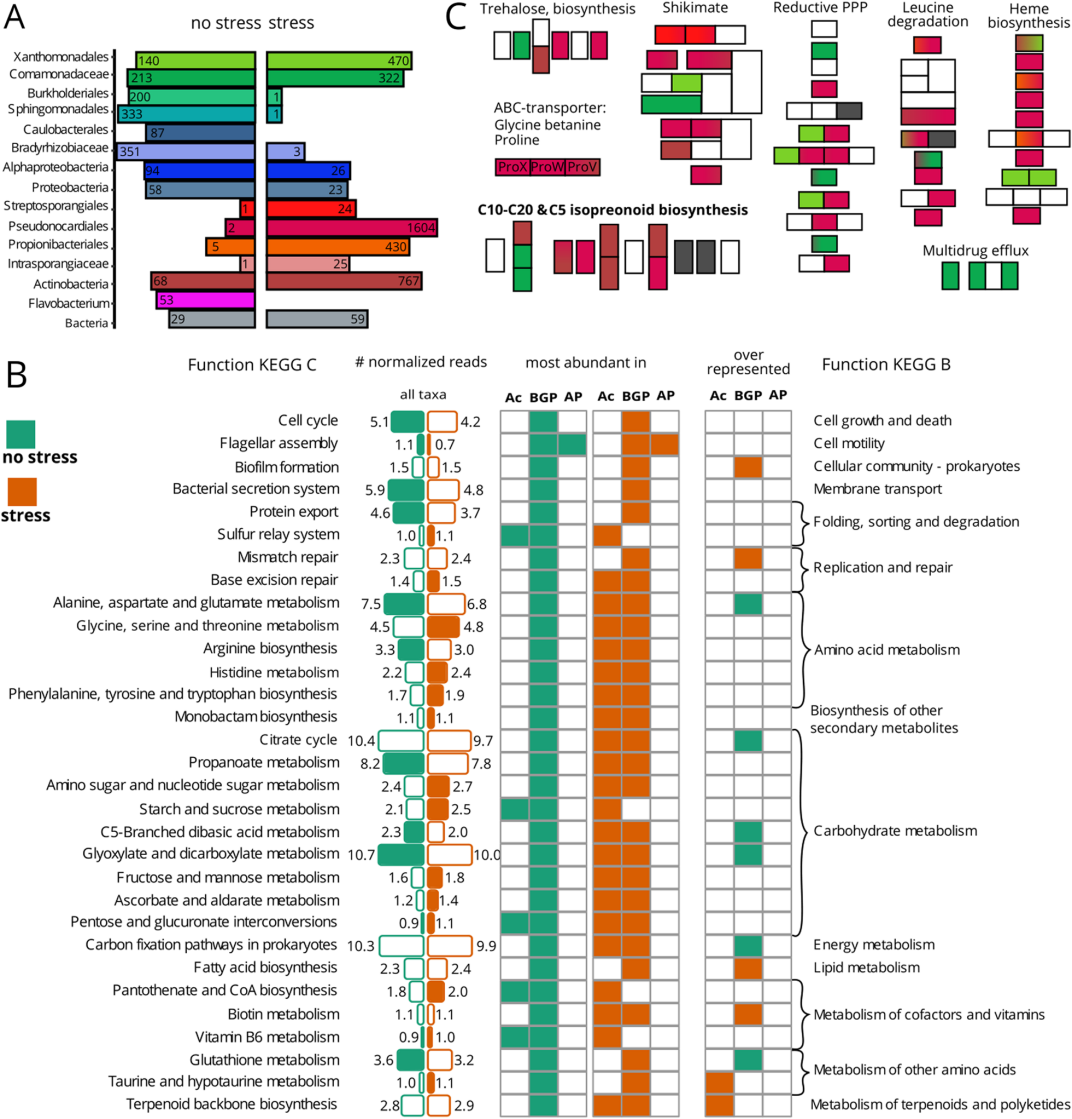
Distinct functions and genes between rhizosphere metagenomes from potato plants cultivated under combined stress and no stress. **A** The number of significant (FDR < 0.05) differently occurring genes per best taxonomic level. **B** Bar sizes show the mean abundance in normalized reads of a function. Filled bars indicate a significant (FDR < 0.01) fold change between stress treatments. Whether a function is most abundant in Actinobacteria (Ac) Beta-Gammaproteobaceria (BGP) or Alphaproteobacteria (AP) is indicated in the first column for non-stressed and in the second column for stressed metagenomes. Comparing the abundance of functions within one taxonomic group revealed weather a function is significantly (FDR < 0.01) overrepresented in rhizosphere metagenomes from potatoes cultivated under stress or no stress. **C** Functional modules that are more abundant in stressed rhizosphere metagenomes: M00121-Heme biosynthesis, M00364 C10-C20 and M00096-C5-isoprenoid synthesis, M00022-Shikimate pathway, M00165 reductive pentose phosphate pathway (PPP), M00698 Multidrug efflux, M00565 Trehalose biosynthesis, M00036 Leucine degradation, phosphotransferase system (PTS). Each block represents a group of KEGG orthologous, for some a gene name is suggested. Colours match the taxa in (**A**), grey boxes were significantly enriched in more than three taxa

### Genotype-specific differences in stressed potato plants

The Bray–Curtis distance showed that the microbial communities were more similar for samples belonging to the same genotype than to different genotypes. Still, the main effect on the microbiota is explained by the applied combined stress conditions (Additional file 1: Fig. S5A). Concordantly, general linear models revealed significant effects for stress and genotype in root and rhizosphere samples (Additional file 1: Table S5). Community structures according to combined stress and genotype were most prominent in the subset of rhizobacteria leading to distinct clusters in the PCoA (Additional file 1: Fig. S5B). Noticeable is the separation of the diploid (Additional file 1: Fig. S5B, dark red and light red) vs. tetraploid (other colours) potatoes under combined stress. The fungal community in roots and rhizosphere as well as the bacterial community in roots were also significantly affected by combined stress and genotype but less profoundly (Additional file 1: Fig. S5, Table S5). In general, the F-value of the factor stress reduced from the rhizosphere to the root microbiota (Additional file 1: Table S5A) indicating a lower stress effect on root microbiota as compared to the rhizosphere. Also, fungi were less affected than bacteria. In contrast, the F-value of the factor genotype was similar between subsets, indicating a constant effect of the genotype on the microbiota (Additional file 1: Table S5).

**Fig. 5:**
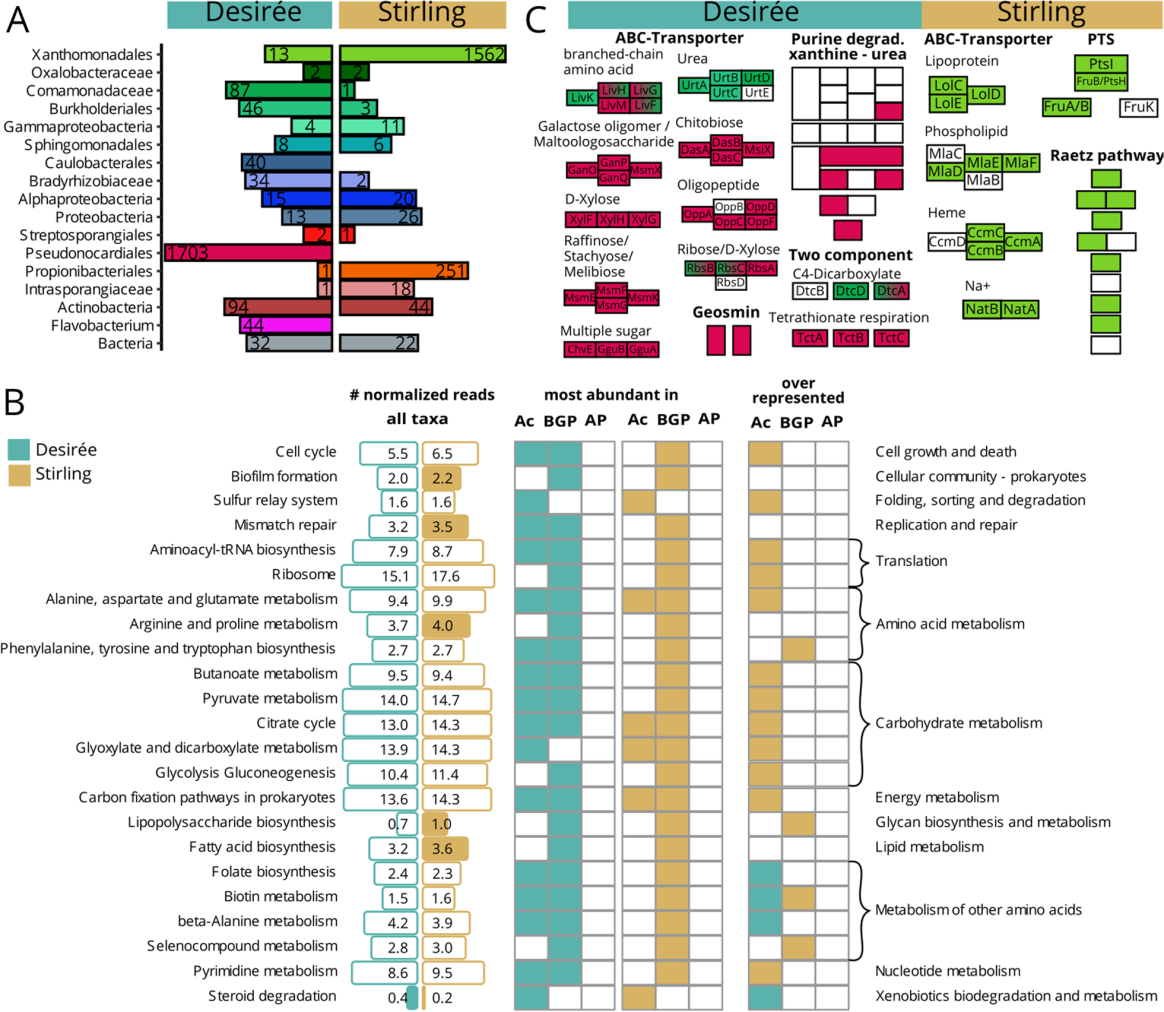
Distinct functions and genes between rhizosphere metagenomes from a good (Stirling, ocher) and poor (Desirée, turquois) performing potato genotype cultivated under combined stress. **A** The number of significant (FDR < 0.05) differently occurring genes per best taxonomic level. **B** Bar sizes show the mean abundance in normalized reads of a function. Filled bars indicate a significant (FDR < 0.01) fold change between potato genotypes. Whether a function is most abundant in Actinobacteria (Ac) Beta-/ and Gammaproteobaceria (BGP) or Alphaproteobacteria (AP) is indicated in the first column for Desirée and in the second column for Stirling metagenomes. Comparing the abundance of functions within one taxonomic group revealed whether a function is significantly (FDR < 0.01) overrepresented in rhizosphere metagenomes from Desirée or Stirling. **C** Functional modules that are distinct between genotypes: M00121-Heme biosynthesis, M00364 C10-C20 and M0546-Purine degradation, M00866 Raetz pathway, phosphotransferase system (PTS). Each block represents a group of KEGG orthologous, for some a gene name is suggested. Colours match the taxa in (**A**)

### Correlating diversity and microbial abundance with stress responses of tetraploid potato plant growth

Different potato genotypes showed different phenotypic stress response patterns including effects on final yield, foliage, half-time to canopy closure, number of young tubers and diameter of the largest young tuber (see also Additional file 1: Fig. S1E,F). The abundance of some ASVs correlated with the phenotypic stress responses of potato plants, which could be grouped in clusters (Fig. 3, Additional file 1: Table S6). For instance, *Xanthomonadales* sp. ASV_465, *Chitinophaga arvensicola* ASV_499 and *Occallatibacter* sp. ASV_869 were more abundant in the rhizosphere of potato genotypes with a stable yield (Fig. 3A, ***, cluster III). In contrast, *Flavobacterium* sp. ASV_30 in the rhizosphere and *Streptomyces sp.* ASV_168 in roots were most abundant in genotypes that suffered from high yield loss under combined stress. The abundance of most microbes correlated highly significantly with a faster half-time canopy closure (Fig. 3A VI, 3C IV, ***). One example is *Dyadobacter* sp. ASV_47 that correlated to half-time canopy closure in root and rhizosphere samples. The fungi *Trichocladium opacum* ASV_44 and *Mortierella hyalina* ASV_24 occurred in the rhizosphere of stress-resilient genotypes and correlated with tuber yield (Fig. 3B, I).

### The rhizosphere metagenomes of potato plants exposed to combined stresses and unstressed conditions have distinct functional potentials

Shotgun metagenomic sequencing revealed a huge impact of combined stress on gene abundance (Additional file 1: Table S7). Of 17,548 genes and gene fragments in the bacterial dataset, 31% were more abundant under stress and 27% were more abundant in no-stress samples. More than 2000 stress indicator genes belonged to *Actinobacteria*, represented by *Pseudonocardiales* and *Propionibacteriales*, whereas 800 genes belonged to Beta- and Gammaproteobacteria represented by *Xanthomonadales* and *Comamonadales* (Fig. 4A). Merging all taxa, we identified 14 functional groups (KEGG C-level, Fig. 4B, details in Additional file 1: Table S8) that were more abundant in rhizosphere metagenomes under combined stress conditions, including (i) sugar-, (ii) amino acid- and (iii) vitamin/cofactor metabolism as well as (iv) base excision repair. Most functions were mainly represented by Beta- and Gammaproteobacteria under conditions without water and P limitation, whereas under combined stress Actinobacteria increased in proportion matching the increase of Actinobacteria under the same conditions in the amplicon dataset. Within Actinobacteria, taurine and hypotaurine metabolism and terpenoid backbone biosynthesis were over-represented in rhizosphere metagenomes under combined stress conditions while within Beta- and Gammaproteobacteria biofilm formation, fatty acid biosynthesis, biotin metabolism and mismatch repair were over-represented functions. Beyond KEGG C-level we identified KEGG modules composed of stress indicator genes (Additional file 1: Table S9) and presented a selection in Fig. 4C. Represented by Actinobacteria, a glycine betaine/proline sugar-ABC-transporter was more abundant under combined stress. Furthermore, under these conditions underlying genes for trehalose biosynthesis were more abundant in rhizosphere metagenomes.

In addition to drought, plants were exposed to P limitation. Concordantly, the OmpR two-component system involved in phosphate assimilation and a phosphate ABC-transporter were more abundant under combined stress but only in Actinobacteria (Additional file 1: Table S9). Also, the heme biosynthesis, pentose phosphate and leucine degradation pathways were more abundant under combined stress (Fig. 4C). Rhizosphere microbiota of stressed plants showed a higher genomic potential to produce (i) isoprenoids (C5 non-mevalonate pathway, C10–C20) and (ii) precursors of aromatic acids and secondary metabolites via the shikimate pathway (Fig. 4C). Summarizing the reads at the higher functional level, KEGG B, revealed an increased abundance of reads assigned to biosynthesis of secondary metabolites such as geosmin (Additional file 1: Table S8). Among the 12 functional groups more abundant in samples from conditions without water and P limitation (Fig. 4B, and more detailed in Additional file 1: Table S8, FDR < 0.01) were cell motility and protein export. Surprisingly, the function glutathione metabolism and four genes similar to the glutathione-S-transferase being involved in detoxification, were more abundant in metagenomes under conditions without combined stress (Additional file 1: Fig. S6B). Within Beta- and Gammaproteobacteria glutathione metabolism was over-represented in samples from conditions without water and P limitation along with carbohydrate metabolism and carbon fixation in prokaryotes (Fig. 4B). Moreover, all five orthologous gene families of urea ABC-transporters were enriched in rhizosphere metagenomes under non-stresses conditions (Additional file 1: Table S9A).

### Functional potential in rhizosphere metagenomes differ between a well and a poorly performing potato genotype

Out of the well and poorly performing genotypes we selected two that grew next to each other, ensuring that they had access to the same pool of soil bacteria for rhizosphere enrichment. Furthermore, we selected Desirée as a poor performer because it is a widely grown cultivar. Desirée produced 7.7 kg tubers under stress, which represents a loss of 55% compared to cultivation conditions without combined stress, while Stirling performed better producing a yield of 9.3 kg, i.e., a loss of only 38%. Under combined stress 1562 genes from Xanthomonadales (Fig. 5A, green) were more abundant in the Stirling rhizosphere metagenomes while 1703 genes from Pseudonocardiales (dark red) were more abundant in the Desirée metagenome (Additional file 1: S10). In samples from the treatment without stress application Xanthomonadales were more abundant in Desirée and Pseudonocardiales more abundant in Stirling (Additional file 1: Fig. S7). Propinobacteriales (more abundant in Stirling) and Flavobacteria (more abundant in Desirée) preferred one genotype regardless of the stress conditions.

Distinct functional groups dominated, i.e., were most abundant in sequence numbers by distinct taxonomic groups (Fig. 5B). In Desirée, Actinobacteria together with Beta- and Gammaproteobacteria dominated most functional groups while in Stirling mainly Beta- and Gammaproteobacteria dominated most functions. More sequences were assigned to lipopolysaccharide biosynthesis in Stirling and the function was over-represented in Beta- and Gammaproteobacteria in the rhizosphere of Stirling compared to Desirée (Fig. 5B). Similarly, biofilm formation and fatty acid biosynthesis via the Raetz pathway, together with genes for ABC-transporters of lipoproteins and lipophospholipids, belonged mainly to Xanthomonadales and were concordantly more abundant in Stirling (Fig. 5C, details: Additional file 1: Tables S11 & S12). Other Stirling-associated Xanthomonadales were, based on their metagenome information, likely involved in (i) the conversion of L-cysteine via glutathione to L-glutamate (glutathione metabolism), (ii) the conversion of taurine to 5-glutamyltaurine (Additional file 1: Table S12E) (iii) fructose uptake (phosphotransferase system, PTS, Fig. 5C) (iv) the production of auxin by the tryptophan 2-monooxygenase (*iaaM*) (Additional file 1: Fig. S8A) and (v) in the type II secretion system (Additional file 1: Table S12E). One rhizosphere MAG MeBa083 was classified as *Lysobacter* (order Xanthomonadales, Additional file 1: Table S4) and contained two bacteriocin-, two lanthipeptide-, one arylpolyene and one polyketide synthase-like region. Carbohydrate metabolism and carbon fixation pathways in prokaryotes were over-represented in Actinobacteria from Stirling compared to Actinobacteria from Desirée rhizosphere metagenomes (Fig. 5B). In total five glutathione-S-transferases from three different taxa were more abundant in Stirling metagenomes (Additional file 1: Fig. S8A).

In rhizosphere metagenomes genes for steroid degradation were more abundant and over-represented within Actinobacteria from Desirée compared to Actinobacteria from Stirling (Fig. 5B). Additionally, folate biosynthesis, biotin and beta-alanine metabolism were over-represented in Actinobacteria from Desirée rhizosphere metagenomes. In general, genes with assigned function that were more abundant in Desirée belonged mainly to Pseudonocardiales (Fig. 5A). Their assigned potential functions included purine degradation to urea and diverse transporter genes for (i) sugars (raffinose, chitobiose, sorbitol, ribose, D-xylose) (ii) oligopeptide and (iii) tetrathionate (Additional file 1: Table S12J). Branched amino acid and C4-dicarboxylate transport genes were from Comamonadaceae and Pseudonocardiales, while more abundant amino acid urea transporter genes were only detected in Comamonadaceae (Fig. 5C). Genes involved in plant growth promotion, such as ACC-deaminase and a pyrroloquinoline quinone biosynthesis gene, were found in Pseudonocardiales (Additional file 1: Fig. S7B). Phosphate ABC-transporter genes were more abundant in the stressed rhizosphere metagenomes of both genotypes: from Pseudonocardiales in Desirée and from Xanthomonadales in Stirling. Biotin metabolism was over-represented in Actinobacteria from Desireé and in Beta- and Gammaproteobacteria from Stirling indicating that different genotype-indicator taxa can have the same function under combined stress.

### Plasmids and phages—mobile elements in potato rhizosphere metagenomes

**Fig. 6:**
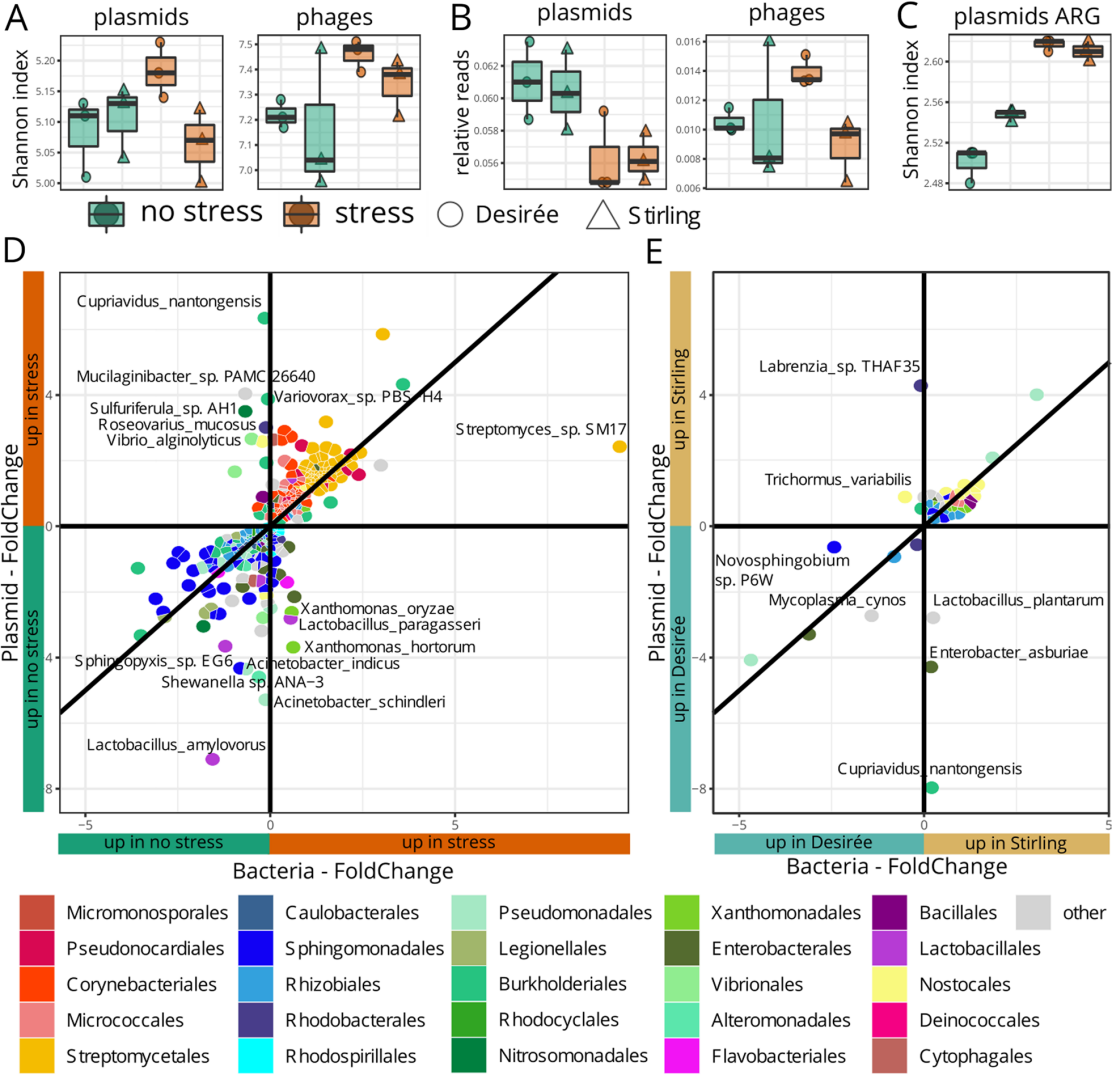
Distinct mobile elements between potato rhizosphere metagenomes. All box plots show the twelve samples of (i) no stress Desiree, (ii) no stress Stirling, (iii) stress Desirée and (iv) stress Stirling by **A** the diversity by Shannon Index **B** the number of relative reads of plasmids and phages of all taxonomic classified reads, and **C** diversity by Shannon Index considering only antibiotic resistance genes (ARG). In **D** and **E** each tile represents a bacterial taxon that is plotted by its plasmids log2 Fold Changes (FC) against the bacteria FC. Only taxa with a significant plasmid-FC are shown (FDR < 0.05). The plasmid and bacterial abundances change in the same ratio for taxa close to the diagonal line, while taxa close to the vertical line have a higher foldchange for plasmids compared to the FC of whole bacteria, indicating important functions on plasmids under distinct **D** stress treatments and **E** stressed rhizospheres of genotypes

Besides functional genes, mobile elements varied between stress treatments: (i) Shannon diversity of phages increased under combined stress (Fig. 6A) and (ii) the relative amount of plasmid sequences was lower in samples under combined stress conditions (Fig. 6B). Interestingly, in rhizosphere metagenomes the Shannon diversity of antibiotic resistance genes on plasmids was higher in stress compared to unstressed conditions (Fig. 6C), indicating a selective advantage of bacterial plasmids harbouring antibiotic resistance genes. Of 7010 phages detected in rhizosphere metagenomes, three were more abundant in non-stress and 49 in stress conditions (Additional file 1: Table S13A). Similarly, of 1535 plasmids detected in rhizosphere metagenomes, 49 were more abundant in non-stress and 104 more abundant in stress conditions (Additional file 1: Table S13B). Noticeably, 68 of the 104 taxa in which plasmids were more abundant under combined stress belonged to *Streptomyces*. Most plasmids changed in the same ratio as bacteria (Fig. 6D, diagonal line) but some plasmids were more abundant in rhizospheres of one of the treatments (with or without combined stress), although the bacterial abundance did not change (Fig. 6D, vertical line).

In addition, we observed differences in mobile elements between the genotypes under stress: (i) the relative number of phages (Fig. 6B) and (ii) the diversity of plasmids (Fig. 6A) was higher in metagenomes of the poorly performing genotype Desirée compared to Stirling. One hundred and six phages and 10 plasmids were more abundant in Desirée rhizosphere metagenomes (Additional file 1: Table S14). Three bacterial taxa, *Cupriavidus nantongensis, Enterobacter asburiae* and *Lactobacillus plantarum*, had a higher portion of plasmids despite minor changes in their whole genomes (Fig. 6E, vertical line). In rhizosphere metagenomes of Stirling compared to Desirée, 37 phages and 67 plasmids were more abundant. This included the plasmids of four *Xanthomonas* species (Additional file 1: Table S14). Two bacterial taxa, *Labrenzia* sp. THAF35 and *Trichormus variabilis* had a higher portion of plasmids despite minor changes in their whole genomes (Fig. 6E, vertical line). But in general, most plasmids were co-enriched with their bacterial hosts (Fig. 6DE, diagonal line). For phages no exact host ID is available.

## Discussion

Due to climate change, reduced use of fertilizers or availability of nutrients combined stress scenarios become increasingly relevant in crop production. Here, we show that for potato combined stress, i.e., water and phosphorus limitation, has a tremendous impact on the potato holobiont at all levels, from microorganisms to phages and from microbial composition to functions, all associated with severe impacts on plant traits. Overall, the responses resemble those typically reported for drought stress suggesting that water limitation had a more severe impact, however, less information is available on the impact of phosphorus limitation (e.g., on microbiomes). Microbial composition and functions in the root environment indicate stress-adapted plant–microbe interactions. Belowground microbes suffer directly from reduced soil moisture as water lacks as a transport medium, solvent, and resource. However, we and Gao et al. [31] found diversity changes due to water limitation (or combined water and P limitation in our study) mainly in the rhizosphere but not in bulk soil samples, indicating an impact of distinct root deposits and exudates on microbial communities under stress. In general, under drought stress monoderm bacteria like Actinobacteria were found to be enriched in most field samples while chaotropic, mobile, diderm bacteria, like Proteobacteria, were found to be reduced [11, 15]. These findings are well in line with those of our study. However, we found that under water and P limitation Xanthomonadales were more abundant in the rhizosphere but not in roots suggesting a compartment-specific adaption to these stress condition. Furthermore, Micrococcales (Actinobacteria) were depleted under combined stress while still most Actinobacteria like *Streptomyces* and Pseudonocardiales were enriched in the rhizosphere.

In roots, the Actinobacteria Propionibacteriales and Streptosporangales were additionally enriched, indicating an adaption to plant metabolism besides e.g., drought resistance of the bacteria by sporulation or thick cell membranes. In contrast to the bacterial root community, combined stress only marginally influenced the fungal root community. One exception was the phylum of the plant pathogens *Olpidium* (depleted in rhizosphere and roots under combined stress) that propagate as motile spores [32]. Also, motile bacteria and their genetic potential for flagella assembly were depleted under combined stress. In the rhizosphere, the fungal taxa Sordariales were favoured while Pleosporales were depleted by combined stress like in grassland soils under drought [33].

Metagenome analysis of the rhizosphere revealed that half of all detected genes was differentially abundant in rhizosphere metagenomes under combined stress and non-stress conditions indicating strong stress effects on microbial functions. Various sugars serve as osmoprotectants, and pathways of ‘fructose-/ mannose metabolism’ and ‘ascorbate- / aldarate metabolism’ were more abundant in rhizosphere metagenomes under stress. The higher abundance of the pathways ‘base extinction repair’ and ‘fatty acid biosynthesis’ under combined stress suggests an adaptation of the microbial community towards a higher tolerance of DNA and membrane damaging agents such as reactive oxygen species (ROS). ROS are detoxicated by antioxidant systems that utilize cofactors, like (i) the pentose phosphate pathway with the potential to maintain NADPH availability (ii) vitamin B6 (pyridoxine) metabolism, (iii) pantothenate and coenzyme A biosynthesis and (iv) heme biosynthesis, which were all enriched functions under the stress condition applied. Furthermore, genes in Beta- and/or Gammaproteobacteria involved in biotin metabolism were enriched. Cofactors are involved in root growth [34] and root colonization [35], defence [36] and may directly promote plant growth by alleviating osmotic and oxidative stresses [37, 38]. The enrichment of diverse cofactors in this study points to their importance for diverse plant–microbe interactions under stress.

We identified more genes involved in the biosynthesis of trehalose under combined water and P limitation, pinpointing to a potential contribution of trehalose-producing bacteria to enhanced plant tolerance of drought [39]. Furthermore, Actinobacteria carrying genes involved in terpenoid backbone biosynthesis were over-represented in rhizosphere metagenomes under stress conditions, suggesting a role of terpenoids in stress mitigation. Besides terpenoid biosynthesis, the potential of terpenoid degradation of steroids was higher in microbial metagenomes under combined stress only in the poorly performing potato cultivar Desirée.

The pathways of sulfonic amino acid taurine metabolism and the sulphur relay system were mainly represented by Actinobacteria and more abundant under combined stress in the rhizosphere. Even within Actinobacteria the taurine metabolism was over-represented in the rhizosphere under stress conditions, indicating the recruitment of Actinobacteria active in sulphur metabolism. This links water or P limitation to sulphur metabolism, as suggested by Kaya et al. [40]. Phosphorus limitations decrease selenate and selenite adsorption of soils [41] and promote selenium uptake in wheat [42]. Interestingly, within Beta-/and Gammaproteobacteria the selenocompound metabolism was over-represented in the rhizosphere of Stirling under stress conditions, indicating that recruitment of Beta- and/or Gammaproteobacteria active in selenocompound metabolism in Stirling rhizospheres might be a reaction to reduced P availability.

### Functions in rhizobacteria match distinct growth strategies of potato genotypes under stress

Canopy cover and tuber bulking of potato plants depend on environmental and genetic factors and influence potato performance under drought [43]. Although a direct impact of root-associated bacteria on root growth [44], maturity shifts [45] and above stress reponses [46] were shown, we are aware that the plant genotype itself with its physiological proporties will substantially impact plant stress response. Nevertheless, it is likely that plant stress response will be additionally influenced by microbial effects or even a plant physiological response might be induced by microorganisms. Here, various bacterial phyla and bacterial or fungal ASVs correlated in distinct genotypes with high and small yield losses as well as other plant traits. Therefore, we assume that microorganisms might have contributed to the minimal delay of the canopy closure of the variety Desirée under combined stress compared to Stirling. Typically involved microbial functions include (i) ACC deaminase enabling the bacteria to degrade the plant stress hormone ethylene and (ii) a gene known as plant growth promotion factor involved in pyrroloquinoline quinone biosynthesis [47], which we found in this study in Pseudonocardiales genomes found in the variety Desirée. Furthermore, the Pseudonocardiales had genes encoding sugar and branched amino acid ABC transporters as well as C4-dicarboxylate two component systems, pinpointing to the uptake of photosynthesis-derived plant exudates [48].

Most microbes in the roots and rhizosphere correlated with only a minimal canopy closure delay but none of those also correlated with genotypes having lower yield losses under combined stress. Only a *Streptomyces* ASV_62 was abundant in the rhizospheres and roots of potato plants with delayed young tuber development under water and nutrient limitation, a trait which correlated with lower yield loss under combined stress. Concordantly in *Arabidopsis thaliana* bacterial inoculation delayed plant development to overcome long-term water deficits [40], indicating that beneficial microbes delaying plant development at early stages under combined stress might be interesting for agricultural application.

In the rhizosphere of the good performer Stirling compared to Desirée we detected more abundant genes from biofilm pathways and modules like lipopolysaccharide production including the Raetz pathway, the transport of lipoproteins and phospholipids [49]. Xanthomonadales are known to produce biofilms in soils and rhizosphere, alone but also in consortia [50, 51]. In this study candidates for multispecies biofilms are the genera *Granulicella*, *Streptomyces* and *Leifsonia* that co-occurred with Xanthomonadales (Fig. 3, cluster III) and were described to produce exopolysaccharides [52] or to be present in biofilms [53]. Sponge-like biofilms can maintain moisture in the rhizosphere under drought [54], may contain antimicrobial substances [49] and facilitate transport of some minerals and nutrients, which could explain the high abundance of Xanthomonadales in the well-performing genotypes in this study. Moreover, Stirling rhizosphere metagenomes hosted genes encoding tryptophan 2-monooxygenase (*iaaM*) from Xanthomonadales potentially involved in plant–microbe interactions [55]. Interestingly, Na et al. [56] found that under drought the slower growing genotype of *Panicum milianceaum* L., just like the slower growing Stirling in this study, harboured more *Lysobacter* (Xanthomonadales) at flowering, indicating a potential role of some Xanthomonadales in stress-induced growth delay. Under conditions without stress, Xanthomonadales were more abundant in the faster growing genotype Desirée, whereas this group was enriched in a stress-dependent manner in the rhizosphere of Stirling.

### The role of mobile genetic elements in rhizobacterial adaptions to combined stress

Plasmids and bacteriophages (phages) in bacteria often harbour operons or genes for virulence, quorum sensing, antibiotic resistance, and secondary metabolism. Mobile elements may be exchanged between bacteria to accumulate in populations if needed, thereby enabling a fast adaption to environmental conditions. However, there are high energy costs to maintain plasmids and the lysis of infected bacteria for lytic phage propagation [30]. While plasmids and phages within plant microbiomes have been rarely described, particularly those involved in drought and nutrient stress tolerance, we found significant differences in plasmids and phages occurring under combined stress and unstressed conditions. Overall, the relative abundance of plasmids was reduced under combined water and P limitation, probably because of the high maintenance costs of plasmids. However, specific plasmids were more abundant under combined stress. These plasmids either multiplied in their parent strain or spread to other bacteria and included for instance plasmids from *Variovorax* sp. and *Cupriavidus* sp*.*, both known for comprising strains which improve tolerance of plants to drought [57, 58]. Strikingly, the plasmids of *Cupriavidus* sp*.* were over-represented in Desirée, indicating a genotype-specific advantage under stress encoded on those plasmids*.* Phages, in contrast to plasmids, increased in relative abundance under stress in a genotype-specific manner. Phages typically undergo two different replication cycles, lytic or lysogenic. In the lytic cycle, phages replicate inside host cells, which results in lysis of the host cell and release of progeny viruses. In the lysogenic cycle, temperate phages integrate in the host chromosome (as prophages) and the lysogenized bacterium becomes immune to further infections by the same virus. Plant microbiota are exposed to stressful conditions such as the presence of toxic compounds (e.g., ROS) when plants are under stress. Such conditions may induce prophages to enter the lytic cycle further inducing microbial and phage community shifts and potential induction of horizontal gene transfer. In the rhizosphere of the good performer Stirling, the suggested biofilm could limit the relative abundance of phages [59]. So far, very little information exists on the role of phages in the plant environment. However, we know from the human gut that phages play a major role in microbiome development and adaptation [60] and think that plant-associated phages merit further investigation to understand their role in microbiome modulation and adaptation.

## Conclusion

Our results pinpoint to distinct functions and taxa in rhizobacteria that match the distinct phenotypic potato stress responses but to which extent they manipulate plant growth or whether they react to the plant-chosen growth strategy remains to be elucidated. Besides inter-taxon stress adaptions, we identified changes in plasmid and phage diversity and relative abundance indicating intra-taxon genome adaptions to combined stress in a genotype-specific manner. Mobile elements act faster on genomes than genome adaption through replication and might play an important role in bacterial stress adaption over the time period of a growing season. In this study potatoes were cultivated under constant but reduced water supply (combined with P limitation), therefore, a slower potato growth increased the total amount of water available over the complete life cycle. If rhizobacteria and phages shape the phenotypic stress pattern of potato plants, engineering root-associated microorganisms and phages could be used to ensure a plant stress response matching the needs and watering regime of the farmer.

## Methods

### Experimental site, set-up, and sampling

On the 1th of May 2018, at the James Hutton Institute in Dundee, Scotland, ten *Solanum tuberosum* genotypes (eight tetraploids belonging to group Tuberosum; two belonging to the diploid group Phureja) were planted in clay soil (edaphic soil factors, April 2018, pH: 6; Lime req, Arable: 2.5t/ha; Lime req, Grass: 0.0 t/ha; Extractable Phosphorus: 12.2 mg/l; Extractable Potassium: 242.0 mg/l; Extractable Magnesium: 174.0 mg/l; Extractable Calcium: 1700 mg/l; Extractable Sodium: 12.80 mg/l; Extractable Sulphur: 1.8 mg/l; Extractable Copper: 20.9 mg/l; Extractable Manganese: 3.9 mg/l; Extractable Boron: 0.83 mg/l; Extractable Zinc: 11 mg/l; Organic Matter, LOI: 7.04%). Per variety a character set is available in the European Cultivated Potato Database (www.europotato.org/). The genotypes were grown in polytunnels (9.2 m width × 100 m length; c.f. [61] and ploidy as well as maturity classes are listed in the Additional file 1: Table S1. Two treatments were established: (i) conventional fertilizer application (Defra RB209, ORIGIN 14-14-21, 1050 kg ha^−1^, N = ammonium nitrate) at planting with supplemental irrigation (two to three 30 min. applications per week) and (ii) conventional fertilizer application but without phosphorus fertilizer and with reduced irrigation. The watering was performed as required and this was based on daily soil moisture measurements made at 100, 200, 300 and 400 mm depth using a Delta T PR2 probe (Delta T Devices, Cambridge, UK) at 20 access tube locations buried throughout the experimental plots. Irrigation was performed at the surface using a drip irrigation system. Probe). Soil moisture values are shown in Additional file 1: Fig. S1C and D. For microbial analysis soil and plant samples were taken from the 7th to 10th July, i.e., after 52 days after planting, at constant warm weather. Each plot consisted of two genotype and two border rows (Additional file 1: Fig. S1A, B). Bulk soil was sampled at random sites between the plots. Out of eight plants per row in a plot, four with a similar growth but not the border plants, were selected for analysis. Using a potato fork, whole plants with root systems were removed and a representative collection of 6–10 dirty but shaken root branches (no stolons) were collected into 50 ml tubes, stored in a cooling box and samples were prepared for DNA extraction at the same day. At destructive microbiome sampling, the largest diameter of each tuber (all called young tubers) was recorded. In an adjacent experiment, the half-time to canopy development as well as yield loss (foliage and tuber; Additional file 1: Fig. S1E,F) were determined.

### Sample preparation and DNA extraction

Soil samples were homogenized and particles larger than 0.5 cm were removed. Tubes containing the roots and 25 ml of sterile water were shaken for 3 min. Centrifugation of the suspension for 10 min at 4000 × g sedimented the rhizosphere soil. Root samples were (i) washed under running tap water, (ii) surface sterilized by submerging them for 5 min in 2.5% NaOCl enriched with one drop of Tween 20, (iii) washed three times in sterile water and (iv) dried in the oven (85 °C) overnight. Cut roots (length 0.5 cm) were frozen at − 80 °C and homogenized twice for 1.5 min in a TissueLyser at 30 Hz in two different orientations. Root powder (40 ± 5 mg), rhizosphere soil (200 ± 50 mg) and bulk soil (250 ± 10 mg) were stored at − 20 °C in aliquots till DNA extraction, which was performed according to the Qiagen DNeasy Power Soil Kit. The 2 ml reaction tubes were shaken for 10 min twice in a TissueLyser at 20 Hz.

### Amplicon and shotgun metagenomic sequencing and data processing

Amplicon library was prepared in a two-step PCR approach according to Samad et al. [62]. The following primers bind to targeted DNA in the first PCR: (i) for bacteria 799f*-illumina* 5′-*TCG TCG GCA GCG TCA GAT GTG TAT AAG AGA CAG* AAC MGG ATT AGA TAC CCK G-3′; 1175r*-illumina* 5′-*GTC TCG TGG GCT CGG AGA TGT GTA TAA GAG ACA G*AC GTC RTC CCC DCC TTC CTC -3′ and (ii) for fungi ITS1f*-illumina* 5′-*TCG TCG GCA GCG TCA GAT GTG TAT AAG AGA CAG CTT GGT CAT TTA GAG GAA GTA A*-3′ and ITS2*-illumina* 5′-*GTC TCG TGG GCT CGG AGA TGT GTA TAA GAG ACA G GCT GCG TTC TTC ATC GAT GC*-3′′. Each sample was processed in (i) four biological replicates and (ii) three technical replicates to (i) consider biological variance and (ii) reduce random PCR effects. Bacterial amplicons of 480 bp from root-DNA were extracted from 2% agarose gels. enriched via gel extraction. In the second PCR we indexed each sample with primers of the Nextera XT Index Kit (Illumina, Inc, USA). A blank DNA-isolation without material and a control library (D6305, ZymoBIOMICS, USA) were included. The samples were sequenced on an Illumina MiSeq at the Competence Unit Bioresources of the AIT Austrian Institute of Technology in Tulln. Illumina MiSeq reads were filtered with Bowtie2 v2.3.4.3 [63] to avoid the presence of Illumina PhiX contamination and quality was preliminarily checked with FastQC v0.11.9 [64]. Primers were stripped using Cutadapt v1.18 [65]. Sequences were quality filtered, trimmed, denoised and amplicon sequence variants (ASVs) were generated with DADA2 v1.14 [66]. Denoised forward and reverse ASV sequences were merged, and chimeras were removed. Filtered ASVs were checked using Metaxa2 v2.2.1 [67] and ITSx v1.1.2 [68], respectively, for targeting the presence of V3-V4 16S rRNA and ITS1 region, in bacterial sequences and fungal sequences. Taxonomic assignment of 16S-rRNA-gene ASVs and ITS-based ASVs was performed using the RDP classifier [69] of DADA2 against the SILVA v138 [70] database and UNITE 8.2 [71] database, respectively. BIOM objects (i.e., count matrices equipped with taxonomic information) with bacterial and fungal counts were built and imported into the R statistical environment.

For shotgun metagenomics the DNA was cleaned following the Microcon Ultracel YM-30 (Merck, Germany) protocol. Using three biological replicates, the Vienna Bio Core Facility prepared the DNA library with the NGS DNA Library Prep Kit (Westburg, Netherlands) and 2 × 150 bp sequencing was conducted on a NovaSeq (Illumina, USA). Illumina’s PhiX reads were filtered out of the sequencing data with Bowtie2 v2.3.4.3 [56]. Filtered reads were processed using fastp v0.20.1 [72] with a cutting-by-quality sliding-window approach from 5´ to tail and 3´ to the front of each read. The selected window size was 4 bp with a minimum quality of Q20. Adapters were auto-detected and removed. A quality check was carried out with FastQC v0.11.9 [64]. Fastp and FastQC output summaries, respectively, were inspected using MultiQC v1.9 [73]. The metagenomic reads were used to generate four different datasets. (i) BIOM tables of archaea, bacteria, fungi, phages and plasmids; (ii) metagenome assembled genomes (MAGs); (iii) abundance table of reads mapping against the antibiotic resistance gene database; and (iv) annotated genes and gene-fragments with their abundance.

Ad (i) In more detail, filtered reads were classified with Kraken2 v2.0.9 [74] (confidence = 0.1) against archaeal, bacterial, and fungal genomes downloaded from the NCBI Reference Sequence Database (RefSeq), using the kraken-build routine. The fungal sequence database was then integrated with all fungal genomes available in GenBank, downloaded using the “ncbi-genome-download” script [75]. The plasmid sequence database was built upon data available from NCBI’s RefSeq repository. Bacteriophage sequences were downloaded from GenBank using the NCBI’s E-utilities [76]. All sequences were downloaded, and databases were built between July and August 2020. Abundance estimation of Kraken2 results was inferred using Bracken v2.6.0 [77] and BIOM tables were generated using the kraken-biom v1.0.1 [78] utility.

Ad (ii) Classified archaeal, bacterial, and fungal reads, respectively, were then assembled using MEGAHIT v1.2.9 [79]. Metagenomic assemblies were performed following the recommended settings for low-depth soil metagenomic data and only assembled contigs with a minimum length of 1000 bp were kept. Contigs were later checked by BLAST search against the entire NCBI nt database (downloaded in August 2020) and hits were processed employing BlobTools v1.1.1 [80]. Gene prediction and annotation of archaeal and bacterial contigs were carried out with MetaProdigal v2.6.3 [81] and Prokka v1.14.5 [82]. Gene prediction of fungal contigs was performed with GeneMark-ES Suite v4.33 [83]. The binning of metagenomic contigs was carried out with MetaBat 2 [84] and MaxBin v2.2.7 [85]. For MetaBat 2, an iterative strategy was adopted by looping the binning with all possible combinations of values for “—maxP” (percentage of contigs for binning) and “—minS” (minimum edge score for binning) in a range of min = 60 and max = 95, with an increment of 5, whereas for the “—maxEdges” parameter (maximum number of edges per node), the values ranged between min = 200 and max = 500, with an increment of 50 at each loop. Each resulting binning set was than evaluated with CheckM [86] by considering a completeness ≥ 50%, a contamination < 10% and total number of bins. For MaxBin, a probability threshold of 0.8 was chosen. For both MetaBat 2 and MaxBin, the minimum required length of each MAG was set to 1500 bp. MetaBat 2 and MaxBin outputs, respectively, were then combined using DAS Tool v1.1.2 [87], with a score threshold of 0.25. Final MAGs quality was then assessed using CheckM and taxonomy classification was assigned with GTDB-Tk v1.3.0 [88].

Ad (iii) The antimicrobial resistance (AMR) reference gene data (PRJNA313047) were downloaded on 11^th^ of June 2020 and sequences were used to build a BLAST database. Magic-BLAST [89] was utilized to map previously classified Kraken-Bracken reads against the AMR database, with a similarity of 99%. Alignment files were processed with SAMtools v1.10 [90] and BamTools v2.5.1 [91] and mapping results were used to build a table for statistical analysis.

Ad (iv) Magic-BLAST [89] was used to map classified reads against a database built upon previously predicted genes from bacterial metagenomic contigs. BAM alignment files were processed, and a table was generated for further analysis. Functional annotation of predicted proteins was carried out with eggNOG v2.0.3 [92] against the eggNOG DB v5.0.1 [93] and the output was filtered as follows: we excluded (i) non-bacterial tax levels (ii) unspecific gene functions (matching to more than three different KEGG orthologous) and (iii) KEGG functions related to human diseases. The annotation of gene clusters for secondary metabolite biosynthesis was performed with antiSMASH v5.2.0 [94].

### Data analysis

Data analysis was done in R studio using the packages phyloseq [95], tidyverse [96] to organize data and vegan [97] and RAM for ecological measurements. Bray–Curtis distance estimated the bacterial community dissimilarities between the individual samples. The resulting beta-diversity was visualized through nonmetric multidimensional scaling (NMDS), principal coordinate analysis (PCoA) or boxplots. A general linear model evaluated the coefficients of stress-treatment, sample type or genotype for the NMDS or PCoA-scores. Analysis of variance (ANOVA) tested the relevance of this model for our data. Species richness corresponds to the number of distinct ASVs and alpha diversity to the Shannon index. Significant differences in the alpha diversity and microbial species richness between sample types were calculated using the Wilcoxon test for *p *value smaller 0.05. For further analyses reproducible reoccurring core microbiota (detected in at least three out of four replicates) were considered. The taxonomic networks determined by metacoder R-package [98] used the Wilcoxon-test for colouring significant differences in taxonomic abundance at each taxonomic rank. Fold changes for ASVs were calculated by DESeq2 [99] and distinct ASVs selected by a false discovery rate (FDR) smaller than 0.05. Core ASVs in samples under stress conditions that correlated in normalized abundance with phenotypic stress responses of tetraploid potatoes were determined via spearman correlations and a *p *value smaller than 0.05. Stress response per genotype represents the percentual change between yield, foliage mass, half-time canopy closure, number young tubers and diameter of largest tuber under stress and non-stress conditions. Abundance tables of the shotgun dataset for taxa, MAGs, antibiotic resistance genes and eggNOG annotated-genes were analysed in the same way as ASV-tables but differentially filtered. We considered taxa that occurred in two out of three replicates, antibiotic resistance genes with a coverage larger than 20% in two out of three replicates, eggNOG gene fragments with a coverage larger than 50% in two out of three replicates. To identify distinct abundances at a higher functional level the associated reads were summarized and used for Foldchange analysis [99]. To identify over-represented functions within one taxonomic group we combined per sample sequences assigned either to (i) Actinobacteria, (ii) Alphaproteobacteria or (iii) Beta-/and Gammaproteobacteria and their lower taxonomic ranks by eggNOG (best taxonomic level). Normalized reads were DESeq2’s median of ratios. In the KEGG-database each pathway consists of several KEGG-orthologues. Distinct abundant pathways were considered if we detected at least five differentially abundant gene-fragments that represented at least five distinct KEGG-orthologues. Additionally, KEGG functions at Level C were removed if less than 20% of known KEGG-orthologues were present in our dataset. All significant differentially abundant assembled genes were assigned to pathways by the web-tool KEGG Mapper-Search & Color Pathway [100] to identify submodules. Analyses were visualized in R by ggforce [101], pheatmat [102], formattable [103] and ggplot2 [104]. Layout was adapted in Inkscape (https://inkscape.org).

## Supplementary Information

**Additional file 1.** Supplementary Information.

## Data Availability

The datasets generated and analysed during the current study are available in the NCBI repository, in the bioproject PRJNA720816.
