# Correction to: Potato root-associated microbiomes adapt to combined water and nutrient limitation and have a plant genotype-specific role for plant stress mitigation

**DOI:** 10.1186/s40793-023-00492-y

**Published:** 2023-04-25

**Authors:** Hanna Faist, Friederike Trognitz, Livio Antonielli, Sarah Symanczik, Philip J. White, Angela Sessitsch

**Affiliations:** 1 Bioresources Unit, AIT Austrian Institute of Technology, Konrad-Lorenz-Straße 24, 3430 Tulln, Austria; 2 Soil Science Department, Research Institute of Organic Agriculture (FiBL), Ackerstraße 113, 5070 Frick, Switzerland; 3 The James Hutton Institute, Invergowrie, Dundee, DD2 5DA UK

**Correction to: Environmental Microbiome (2023) 18:18** 10.1186/s40793-023-00469-x

Following publication of the original article [1], it was brought to the authors’ attention that certain text elements intended to guide the reader through the figures had been lost during production of the article.

In Fig. 3, three headers were missing: “A rhizosphere bacteria”, “B rhizo fungi” and “C root bacteria”. Roman numbers for referring to a specific cluster were missing in the originally published figure. At the bottom of the figure, a short explanation of the correlation coefficient had been omitted.

**Fig. 3:**
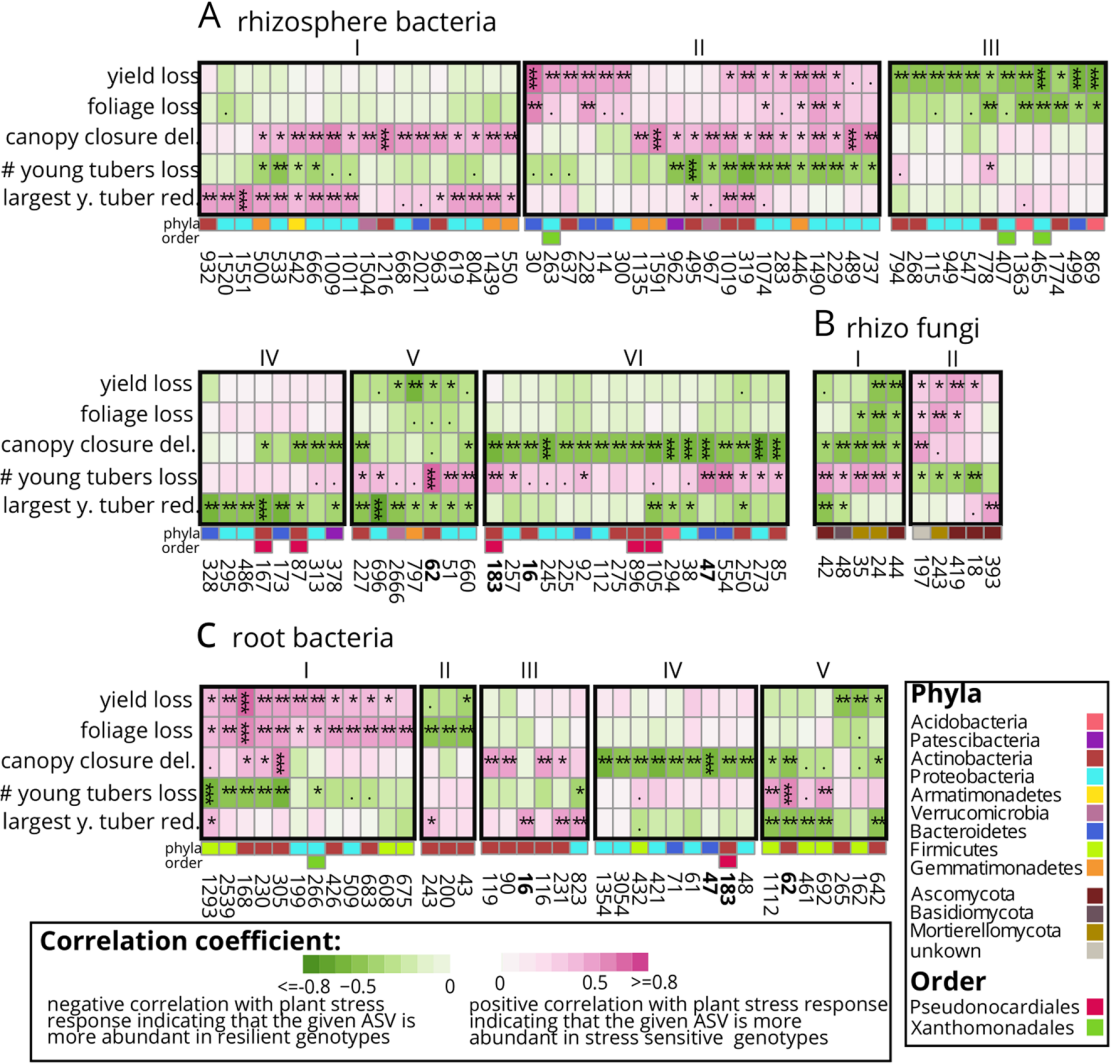
Key microbiota (**A** rhizosphere bacteria; **B** rhizosphere fungi; **C** root bacteria) in stress resilient and suffering genotypes. Columns represent microbes, which are numbered according to their amplicon sequencing variant (ASV) and taxonomical position classified in Additional file 1: Table S8. Rows indicate the strength of potato plant stress responses. This includes percentual tuber yield loss, weight of foliage loss, delay (del.) of half-time canopy closure, differences in number (#) of young tubers and differences in the diameter of the largest young (y.) tuber. Dark pink indicates a high abundance of a specific ASV under stress in potato plants suffering in this phenotype, while green indicates a high abundance in potato plants resilient in this phenotype. Black frames with Roman numbers, indicate clusters of ASVs correlating with the same stress response pattern. Bold ASV-numbers refer to ASVs that correlate with a stress response in roots and rhizosphere samples. Significant spearman correlations are determined by a t-test and results are indicated: *p *value < 0.001: ***p val < 0.01:;**p val < 0.05:;*p  val < 0.1

In Fig. 4A, the elements “stress” and “no stress”, for indicating the treatment in which the genes are more abundant, were missing. Similarly, in **B**, the same text elements had been lost in the left part of the figure, in which green indicated “no stress” and orange “stress”. Moreover, the headers above the columns were missing. From left to right: “Function KEGG C”, “# normalized reads—all taxa”, “most abundant in—Ac BGP AP–Ac BGP AP”, “overrepresented—Ac BGP AP” and “Function KEGG B”. In **C**, the pathway labels were missing. From left to right and top to bottom: “Trehalose, biosynthesis”, “Shikimate”, “Reductive PPP”, “Leucine degradation”, “Heme biosynthesis”, “ABC-transporter”, “C10–C20 & C5 Isoprenoid biosynthesis” and “Multidrug efflux”.

**Fig. 4:**
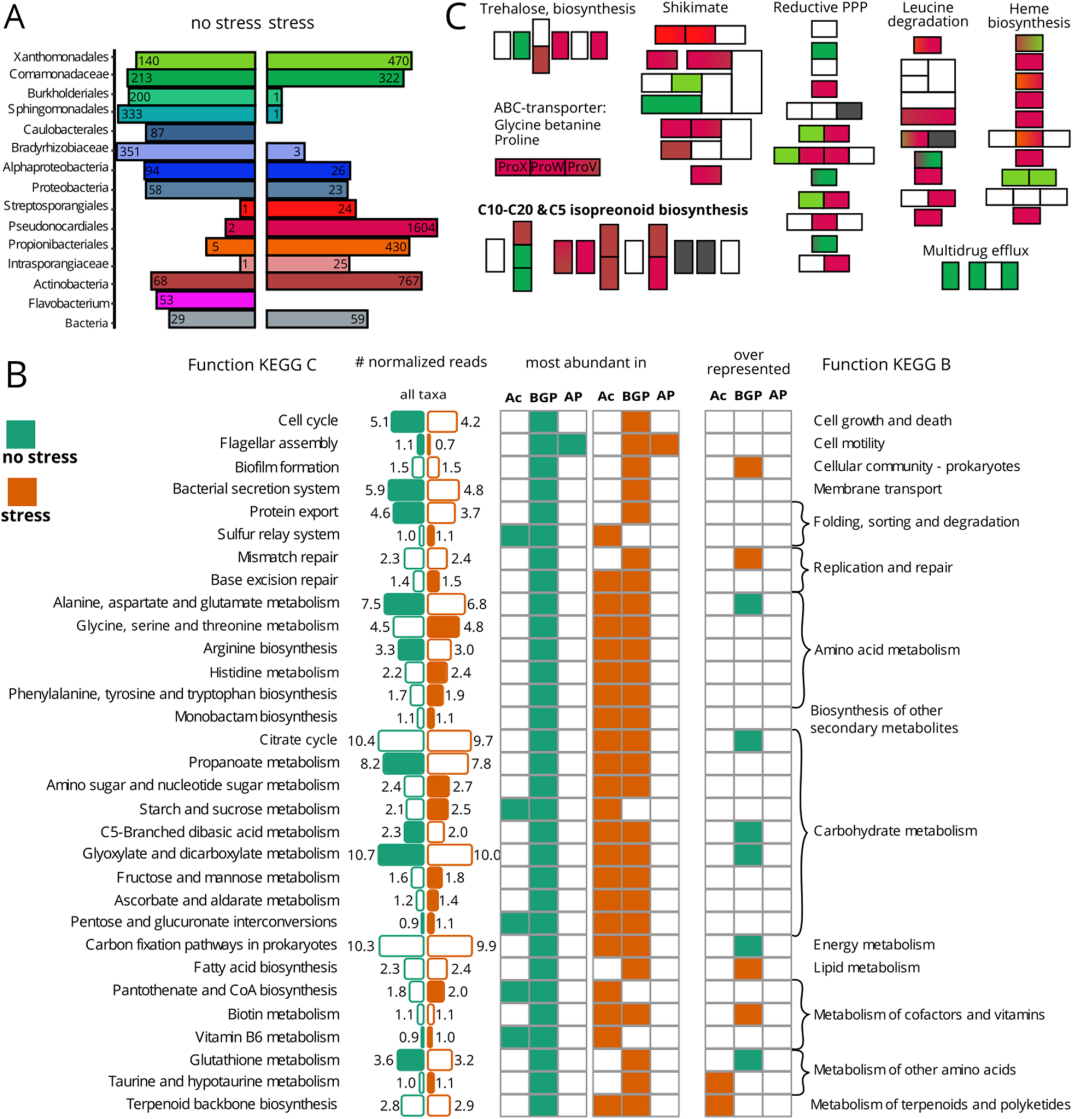
Distinct functions and genes between rhizosphere metagenomes from potato plants cultivated under combined stress and no stress. **A** The number of significant (FDR < 0.05) differently occurring genes per best taxonomic level. **B** Bar sizes show the mean abundance in normalized reads of a function. Filled bars indicate a significant (FDR < 0.01) fold change between stress treatments. Whether a function is most abundant in Actinobacteria (Ac) Beta-Gammaproteobaceria (BGP) or Alphaproteobacteria (AP) is indicated in the first column for non-stressed and in the second column for stressed metagenomes. Comparing the abundance of functions within one taxonomic group revealed weather a function is significantly (FDR < 0.01) overrepresented in rhizosphere metagenomes from potatoes cultivated under stress or no stress. **C** Functional modules that are more abundant in stressed rhizosphere metagenomes: M00121-Heme biosynthesis, M00364 C10–C20 and M00096-C5-isoprenoid synthesis, M00022-Shikimate pathway, M00165 reductive pentose phosphate pathway (PPP), M00698 Multidrug efflux, M00565 Trehalose biosynthesis, M00036 Leucine degradation, phosphotransferase system (PTS). Each block represents a group of KEGG orthologous, for some a gene name is suggested. Colours match the taxa in (**A**), grey boxes were significantly enriched in more than three taxa

In Fig. 5A, the elements “Desireé” and “Stirling”, for indicating the potato genotypes, were missing. In **B**, the headers above the columns were missing. From left to right: “# normalized reads—all taxa”, “most abundant in—Ac BGP AP–Ac BGP AP” and “overrepresented—Ac BGP AP”. In, **C** the headers “Desirée” and “Stirling” and pathway labels were missing. Below the turquois box, the labels were: “ABC-Transporter”, “branched-chain amino acid”, “Urea”, “Glucose oligomer / Maltoologosaccharide”, “Chitobiose”, “D-Xylose”, “Oligopeptide”, “Raffinose/Stachyose/ Melibiose”, “Ribose/D-Xylose”, “Multiple sugar”, “Purin degrad. Xanthine- urea”, “Two component”, “C4-Dicarboxylate”, “Tetrathionate respiration”, “Geosmin”. Below the ocher box: “ABC-Transporter”, “Lipoprotein”, “Phospholipid”, “Heme”, “Na+”, “PTS”, “Raetz pathway”. Within the boxes of each pathway, some abbreviations for genes had been lost.

**Fig. 5:**
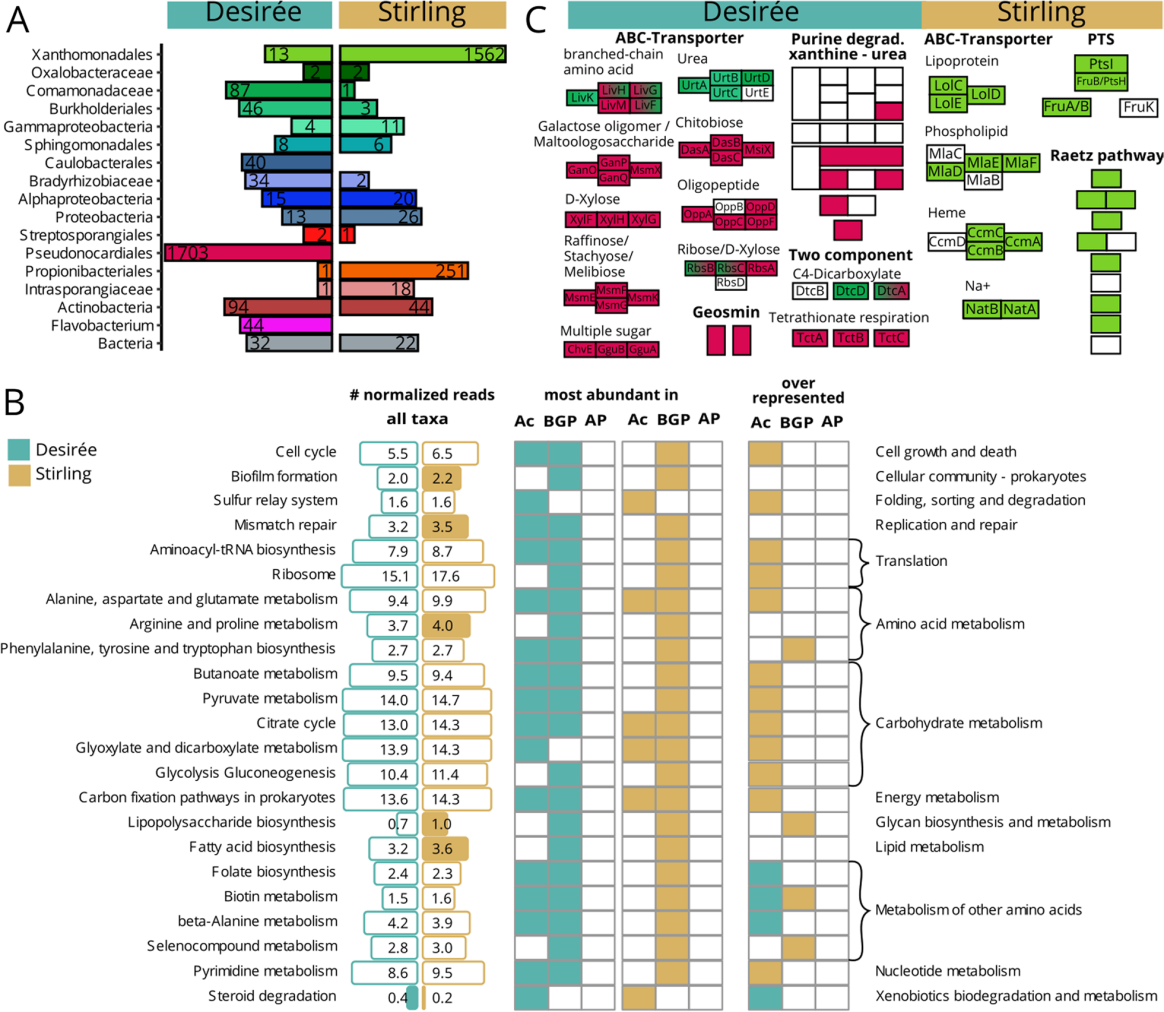
Distinct functions and genes between rhizosphere metagenomes from a good (Stirling, ocher) and poor (Desirée, turquois) performing potato genotype cultivated under combined stress. **A** The number of significant (FDR < 0.05) differently occurring genes per best taxonomic level. **B** Bar sizes show the mean abundance in normalized reads of a function. Filled bars indicate a significant (FDR < 0.01) fold change between potato genotypes. Whether a function is most abundant in Actinobacteria (Ac) Beta-/ and Gammaproteobaceria (BGP) or Alphaproteobacteria (AP) is indicated in the first column for Desirée and in the second column for Stirling metagenomes. Comparing the abundance of functions within one taxonomic group revealed whether a function is significantly (FDR < 0.01) overrepresented in rhizosphere metagenomes from Desirée or Stirling. **C** Functional modules that are distinct between genotypes: M00121-Heme biosynthesis, M00364 C10-C20 and M0546-Purine degradation, M00866 Raetz pathway, phosphotransferase system (PTS). Each block represents a group of KEGG orthologous, for some a gene name is suggested. Colours match the taxa in (**A**)

In Fig. 6A, the y-axis label “Shannon Index” and the headers “plasmids” and “phages” had been missed. In **B**, the y-axis label “relative reads” and the headers “plasmids” and “phages” were missing. In **C**, the y-axis label “Shannon index” and the header “plasmids ARG” were missing. In **D**, the x-axis label “Bacteria—FoldChange” and the y-axis label “Plasmid—FoldChange” were likewise missing. Moreover, “up in no stress” had been written in the green boxes and “up in stress” in the orange ones. In **E**, the x-axis label “Bacteria—FoldChange” and the y-axis label “Plasmid—FoldChange” were missing. Finally, “Desirée” had been written in the turquoise boxes and “Stirling” in the ocher boxes.

**Fig. 6:**
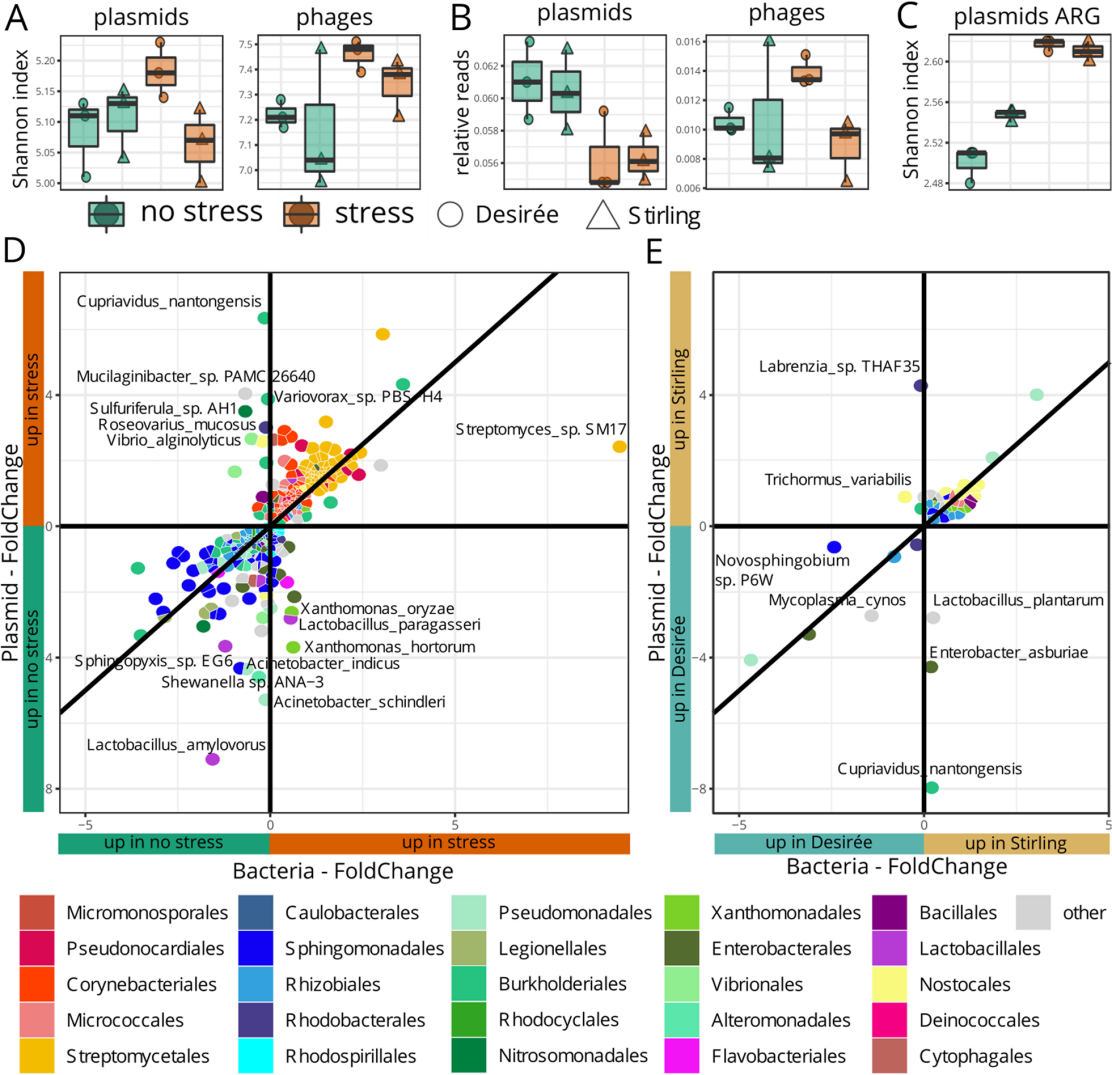
Distinct mobile elements between potato rhizosphere metagenomes. All box plots show the twelve samples of (i) no stress Desiree, (ii) no stress Stirling, (iii) stress Desirée and (iv) stress Stirling by **A** the diversity by Shannon Index **B** the number of relative reads of plasmids and phages of all taxonomic classified reads, and **C** diversity by Shannon Index considering only antibiotic resistance genes (ARG). In **D** and **E** each tile represents a bacterial taxon that is plotted by its plasmids log2 Fold Changes (FC) against the bacteria FC. Only taxa with a significant plasmid-FC are shown (FDR < 0.05). The plasmid and bacterial abundances change in the same ratio for taxa close to the diagonal line, while taxa close to the vertical line have a higher foldchange for plasmids compared to the FC of whole bacteria, indicating important functions on plasmids under distinct **D** stress treatments and **E** stressed rhizospheres of genotypes

The error has since been corrected in the original article.

The authors apologize for any inconvenience caused.
