# Supplementary material for: Potato root-associated microbiomes adapt to combined water and nutrient limitation and have a plant genotype-specific role for plant stress mitigation
Source: Environ Microbiome. 2023 Mar 14;18:18. doi: 10.1186/s40793-023-00469-x (PMC10012461; doi:10.1186/s40793-023-00469-x)
Supplement: Supplementary file 1 — Additional file 1. Supplementary Information. [file 40793_2023_469_MOESM1_ESM.docx]

# Supplemental figures

## Supplemental Figure 1


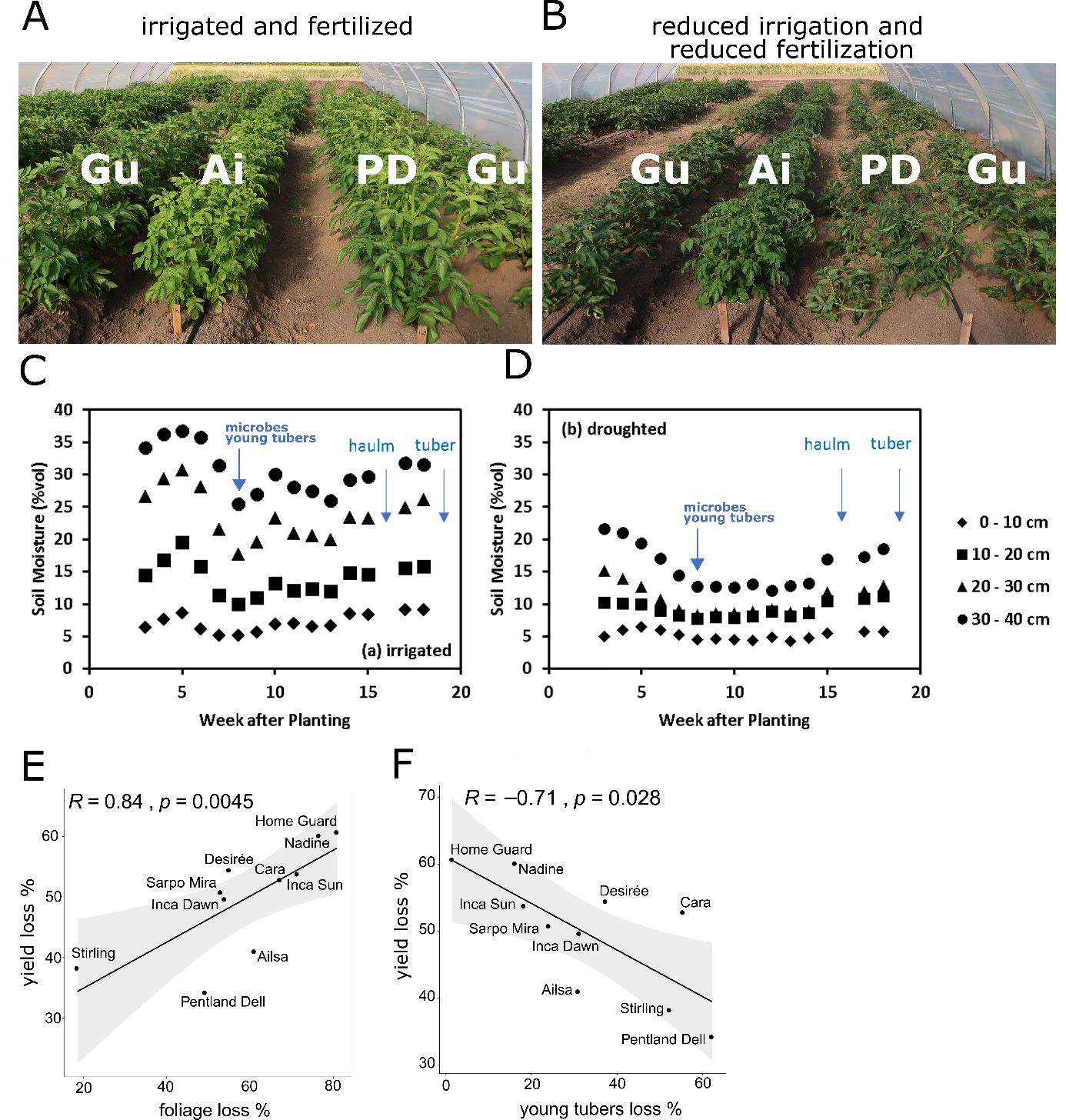


**Supplemental Figure 1.** Combined stress treatment with ten potato genotypes. Experimental site of potatoes growing in polytunnels with sufficient (A) and reduced (B) irrigation and fertilizers. Plots are framed by guard plants (Gu) and visible genotypes are Ai=Ailsa and PD=Pentland Dell. Soil moisture was monitored and higher in irrigated (C) soil as compared to reduced irrigated (D) treatment throughout the experiment showing continuous reduced water availability. The time points of sampling are indicated. Percentual yield loss increased with foliage loss (E) and reduced with young tuber loss (F).

## Supplemental Figure 2


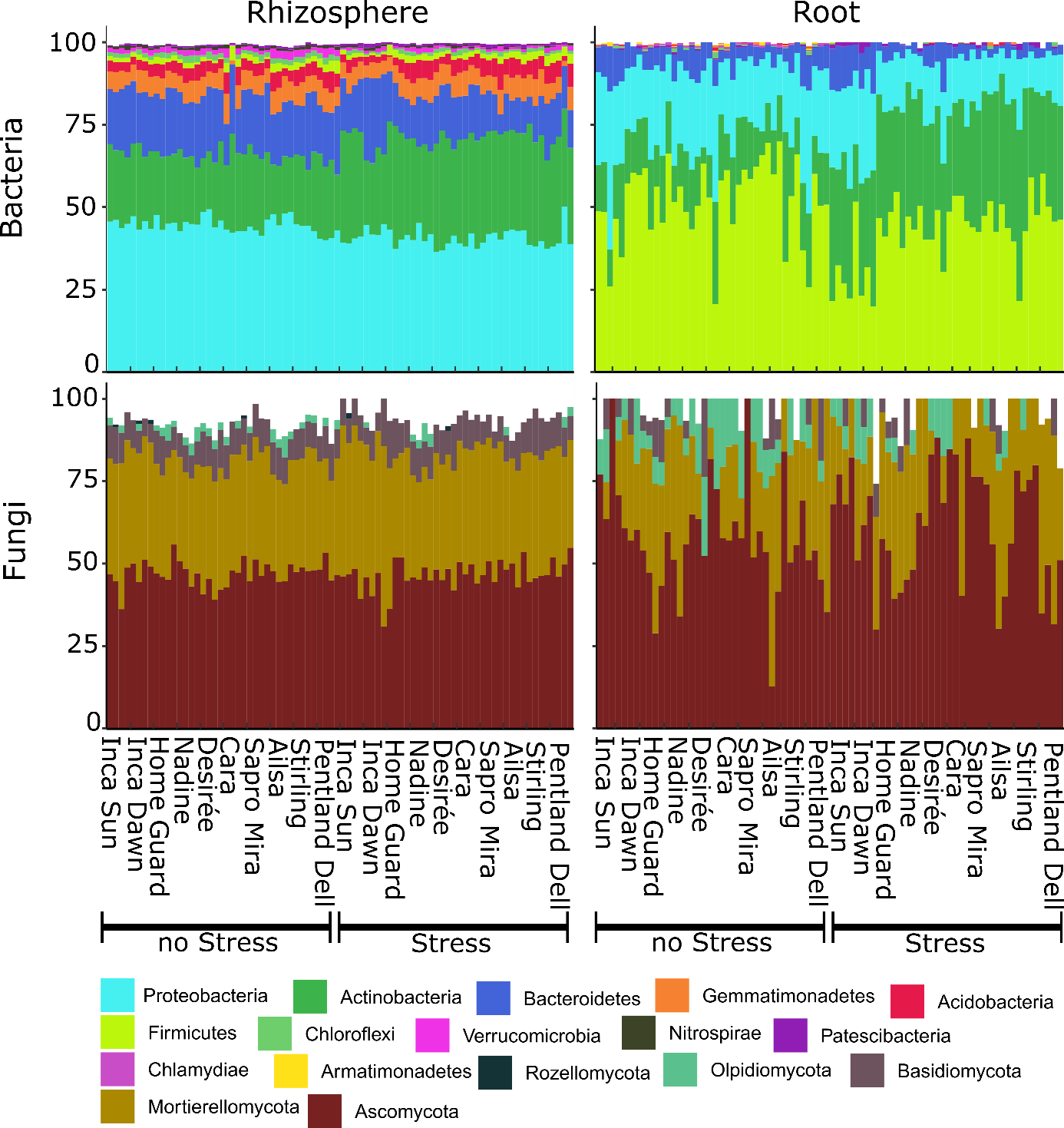


**Supplemental Figure 2**. Relative abundance of the top 10 phyla per sample. Four root samples were dismissed due to insufficient sequencing depth.

## Supplemental Figure 3


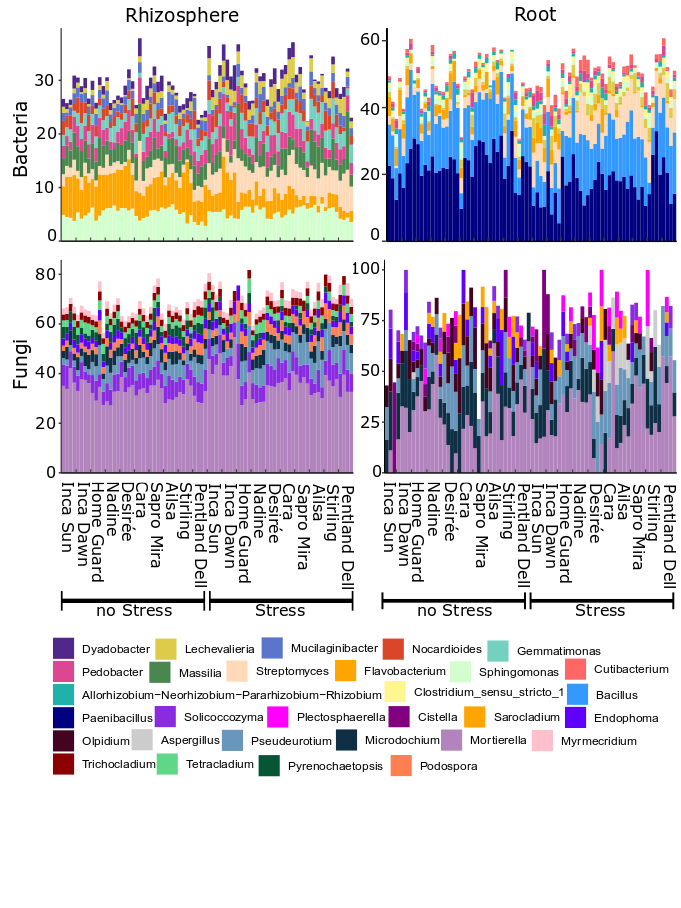


**Supplemental Figure 3**. Relative abundance of the top 10 genera per sample. Four root samples were dismissed due to insufficient sequencing depth. The scale on the y axis differs to increase the resolution per bar chart.

## Supplemental Figure 4


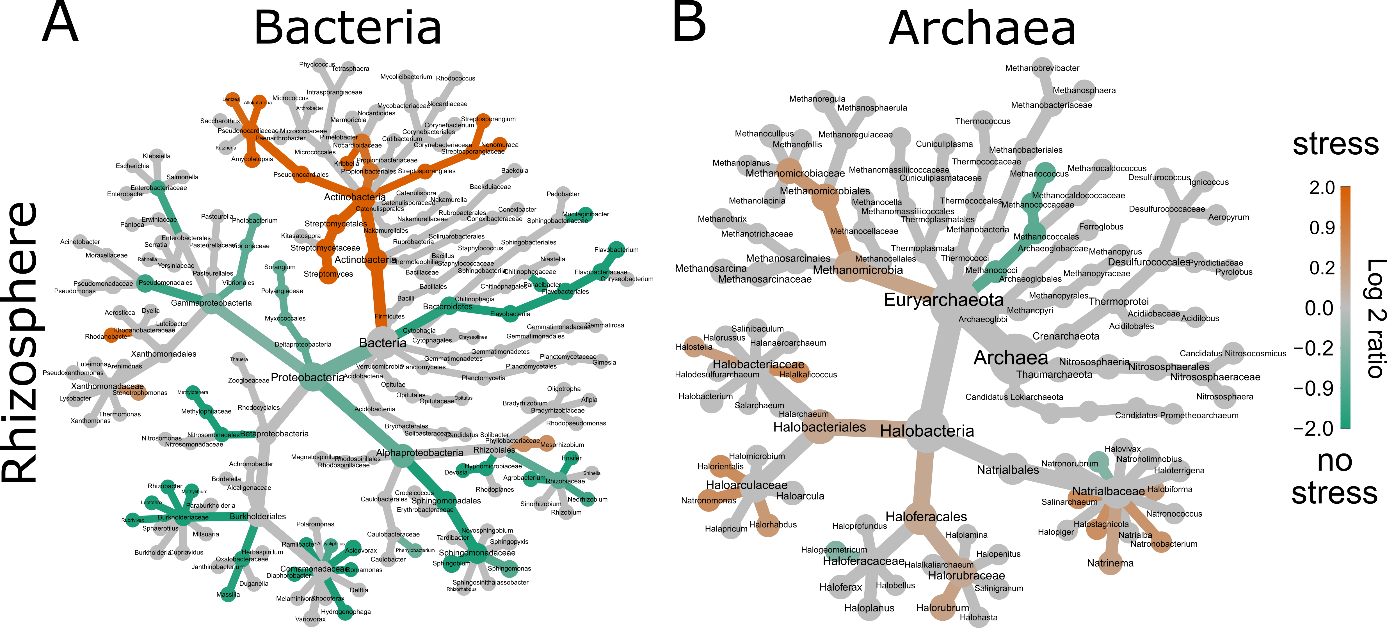


**Supplemental Figure 4.** Changes in microbial composition according to stress treatment based on shotgun metagenome data. Each node represents a taxonomic rank of the reduced shotgun metagenome dataset. Different taxonomic ranks are shown, starting from the highest rank (largest grey nodes) to the genus level at the end of the branches. Coloured nodes are significantly enriched (orange) or reduced (green) under stress (Wilcoxon test <=0.05).

## Supplemental Figure 5


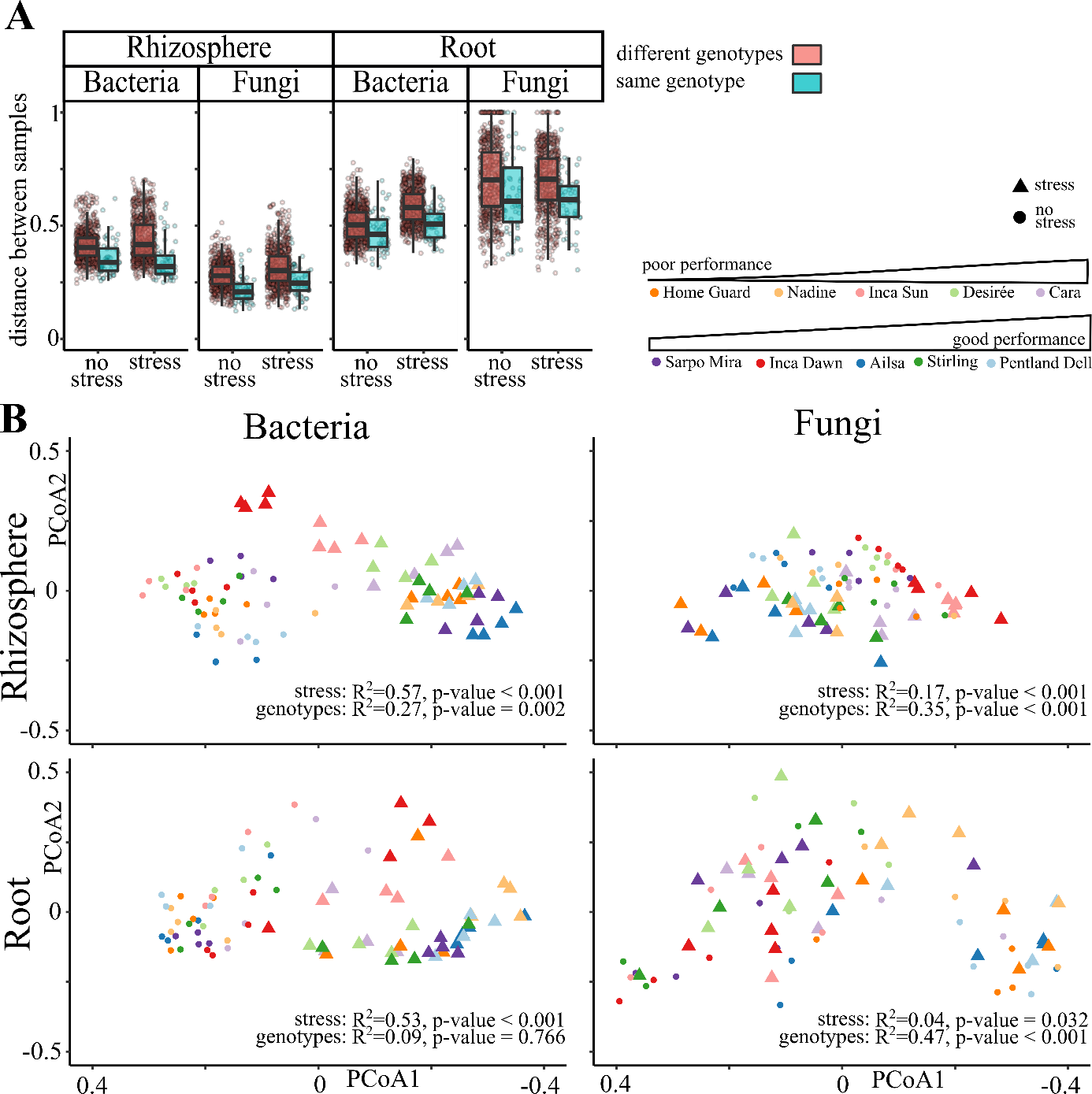


**Supplemental Figure 5**. Potato genotypes host different microbial communities. A) The Bray Curtis distances between two samples from different genotypes (blue) are higher than the distances between two samples from the same genotype (red) for all stress conditions, sample types and in the bacterial as well as the fungal communities. A higher distance indicates a more dissimilar microbial community. B) Split into four datasets (clockwise: Rhizosphere-Bacteria, Rhizosphere-Fungi, Root-Bacteria, Root-Fungi) the principal component analysis scales the Bray Curtis distance between samples onto two axes. Every symbol represents a sample (triangle=stressed sample, point=non-stressed, color=genotype), and samples with similar bacterial community clusters.

## Supplemental Figure 6


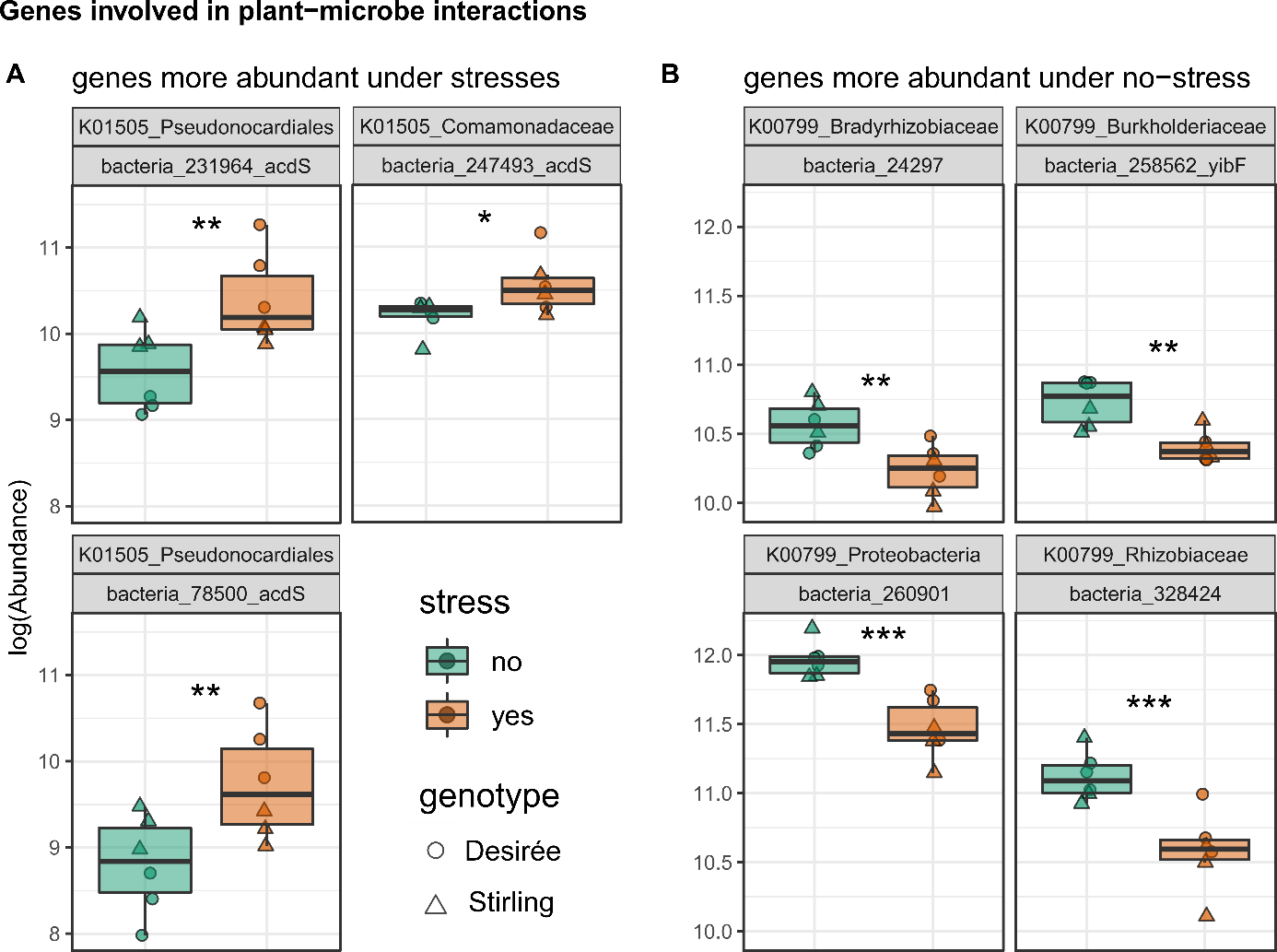


**Supplemental Figure 6.** Genes involved in plant-microbe interactions that show significantly different abundances in rhizosphere metagenomes from stressed and non-stressed potato plants. The first grey box contains the KEGG orthologous group and the best assigned taxonomic level. The second box contains the identification number of the assembled gene and, if available, the preferred gene name. The 1-aminocyclopropane-1-carboxylate (ACC) deaminase (*acdS*, part of K01505) reduces ethylene production, a plant stress hormone. The glutathione-S-transferase is part of the orthologous group K00799 and is involved in reduction of reactive oxygen species. Significance was calculated by a Wald test and corrected to the false discovery rate (FDR) as implemented in DESeq2: FDR<0.05:*; FDR<0.01:**; FDR<0.001: ***.

## Supplemental Figure 7


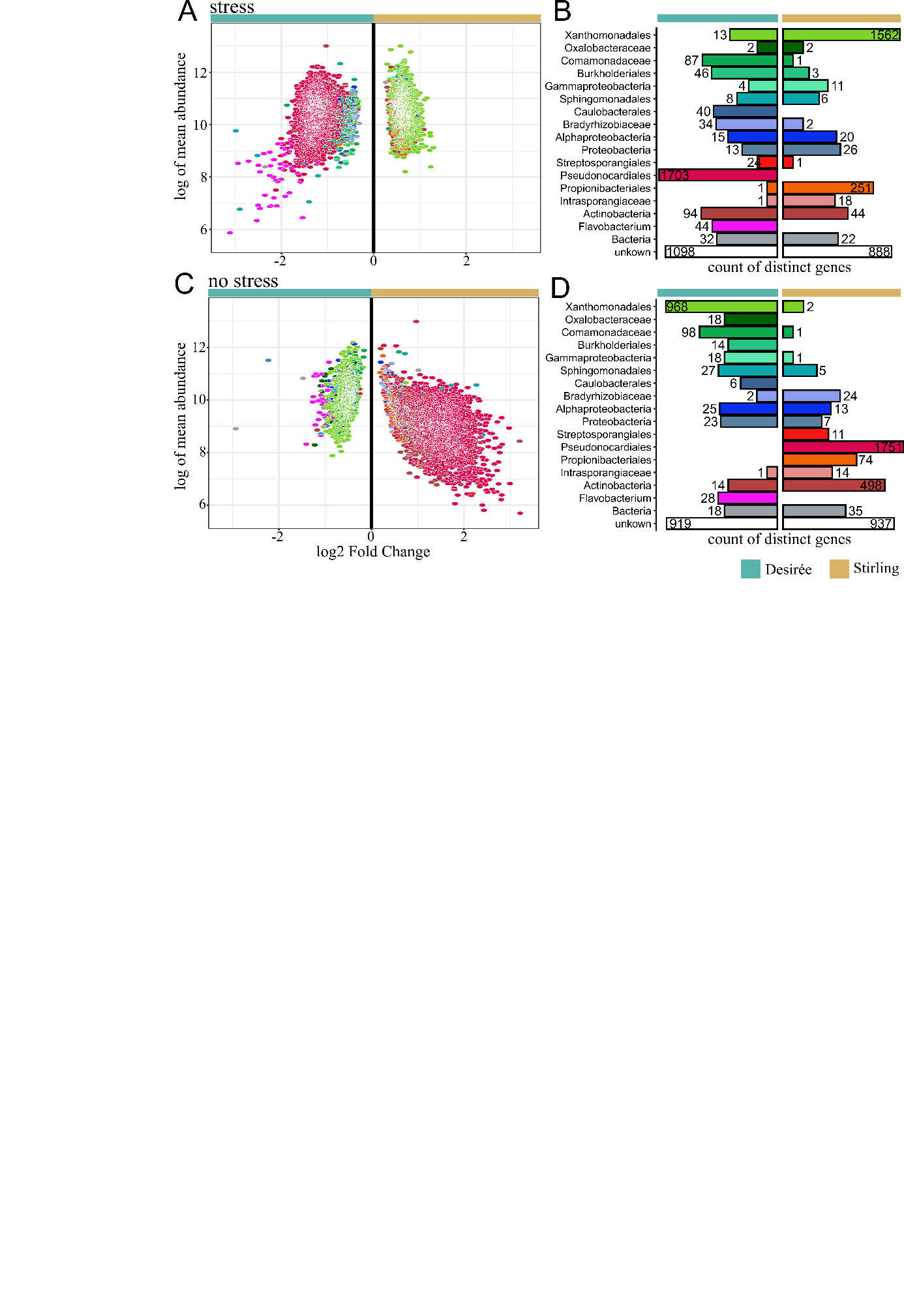


**Supplemental Figure 7.** Distinct genes in rhizosphere metagenomes between Desiree (high yield loss, turquoise) and Stirling (stable yield, ocher) cultivated under combined stresses (A,B) and no stress(C,D). In A,B each tile represents a gene while in B,D the logarithmic size of the bars indicates the number of distinct genes. The colors in A, C match the colors of the taxa in B and D. Only genes with an FDR<0.05 are shown. For further information, genes are listed in Supplemental Table 11.

## Supplemental Figure 8


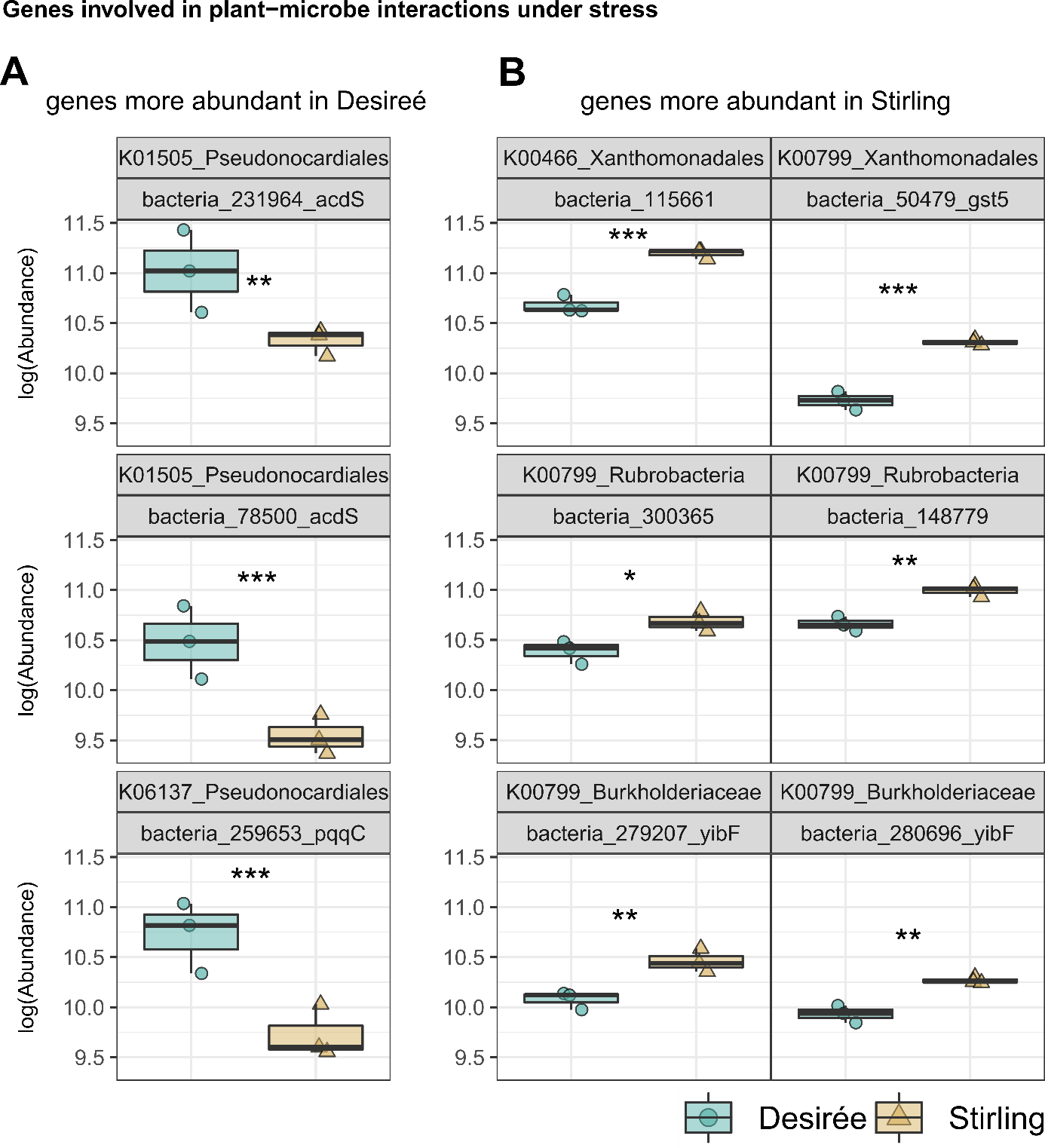


**Supplemental Figure 8.** Genes involved in plant-microbe interactions showing significantly different abundances in rhizosphere metagenomes from Desiree (high yield loss) and Stirling (stable yield) cultivated under combined stress. The first grey box contains the KEGG orthologous group and the best assigned taxonomic level. The second box contains the identification number of the assembled gene and, if available, the preferred gene name. The 1-aminocyclopropane-1-carboxylate (ACC) deaminase (*acdS*, part of K01505) reduces ethylene production, a plant stress hormone. Pyrroloquinoline quinone (part of the orthologous group K06137) and the glutathione-S-transferase (part of the orthologous group K00799) are involved in reduction of reactive oxygen species. Tryptophan 2-monooxygenase encoded by *iaaM* (part of K00466) is a key enzyme in auxin, a plant growth hormone, production. Significance was calculated by a Wald test and corrected to the false discovery rate (FDR) as implemented in DESeq2: FDR<0.05:*; FDR<0.01:**; FDR<0.001: ***.

# Supplemental tables

## Supplemental Table 1

**Supplemental Table 1**: Properties of the analysed genotypes. Yield under no stress and combined stress conditions were compared.

| **Ploidy** | **Variety** | **Yield loss % ± sd** | **Maturity** |
| --- | --- | --- | --- |
| Diploid Phureja | Inca Dawn (DB375/1) | 48±21 | Very late |
| Diploid Phureja | Inca Sun (DB378/1) | 53±7 | Very Late |
| Tetraploid | Home Guard | 61±7 | Very early / early |
| Tetraploid | Nadine | 56±18 | Early |
| Tetraploid | Ailsa | 38±13 | Intermediate |
| Tetraploid | Pentland Dell | 32±17 | Intermediate |
| Tetraploid | Desiree | 52±16 | Intermediate / late |
| Tetraploid | Stirling | 37±12 | Intermediate / late |
| Tetraploid | Cara | 53±5 | Very Late |
| Tetraploid | Sarpo Mira | 51±8 | Very Late |

## Supplemental Table 3

**Supplemental Table 3**. Metagenome assembles genomes from the shotgun dataset. MAGs with a completeness larger 50% and a contamination below 10% are considered.

|  |  | **Quality values** | | **Genome properties** | | | |
| --- | --- | --- | --- | --- | --- | --- | --- |
|  |  |  |  |  |  |  |  |
| **ID** |  | **Complete-**  **ness** | **Conta-**  **mination** | **Genome size**  **(bp)** | **#**  **contigs** | **N50**  **(contigs)** | **# predicted**  **genes** |
|  |  |  |  |  |  |  |  |
| MaBi320 |  | 89.94 | 2.34 | 5229558 | 2156 | 2843 | 6016 |
| MaBi322 |  | 85.04 | 6.44 | 1814010 | 817 | 2497 | 2264 |
| MaBi348 |  | 82.46 | 1.28 | 1779234 | 705 | 3011 | 2162 |
| MeBa003 |  | 67.43 | 3.41 | 2043193 | 684 | 3135 | 2272 |
| MeBa028 |  | 82.82 | 5.72 | 2825538 | 841 | 3774 | 3309 |
| MeBa045 |  | 52.19 | 0.2 | 1037172 | 364 | 2931 | 1194 |
| MeBa057 |  | 76.64 | 3.56 | 4399772 | 1302 | 3728 | 5134 |
| MeBa079 |  | 81.56 | 2.28 | 3895056 | 833 | 5233 | 3901 |
| MaBi162 |  | 73.95 | 6 | 1784871 | 997 | 1813 | 2227 |
| MaBi325 |  | 57.72 | 2.38 | 1558221 | 1006 | 1499 | 1980 |
| MaBi346 |  | 79.34 | 7.64 | 1928298 | 954 | 2196 | 2473 |
| MeBa004 |  | 79.82 | 8.02 | 1936912 | 601 | 3428 | 2305 |
| MeBa052 |  | 72.55 | 5.79 | 2015278 | 593 | 3724 | 2355 |
| MeBa054 |  | 53.32 | 9.58 | 2114883 | 894 | 2384 | 2569 |
| MeBa059 |  | 51.28 | 0.92 | 952062 | 370 | 2612 | 1166 |
| MeBa083 |  | 54.56 | 4.7 | 3527859 | 1572 | 2212 | 4070 |
| MeBa094 |  | 61.23 | 7.33 | 1038318 | 378 | 2861 | 1305 |

# = number, N50 = size of the smallest contig that is needed to sum up 50% of the genome

## Supplemental Table 4

**Supplemental Table 4**. Abundance and Fold Change (FC) of Metagenome assembles genomes (MAGs) in stressed vs non-stressed samples. Analysis was performed using DESeq2 based on negative binominal general linear model fitting and a Wald significant test for p-value calculation. The p-value was adjusted according to Benjamin-Hochberg, resulting in the False Discovery Rate (FDR). Highlighted are significantly different abundant MAGs (blue, FDR < 0.001), enriched MAGs in stressed samples (FC>0, orange) and enriched MAGs in non-stressed samples (FC<0, green).

| **classification** | | | **mean sequence number** | | **fold change (A/B)** | | **statistics** |
| --- | --- | --- | --- | --- | --- | --- | --- |
|  |  |  |  |  |  |  |  |
| **ID** | **order** | **genus** | **A (stress)** | **B (no stress)** | **log2**  **FC** | **log2FC SE** | **FDR** |
|  |  |  |  |  |  |  |  |
| MeBa052 | Sphingobacteriales | *Pedobacter* | 2.5E+05 | 2.4E+05 | **1.2** | 0.8 | 2E-01 |
| **MeBa057** | Streptosporangiales | *Nonomuraea* | 1.2E+07 | 9.1E+06 | **0.4** | 0.0 | 2E-21 |
| **MaBi320** | Mycobacteriales | *Amycolatopsis* | 1.3E+07 | 1.0E+07 | **0.4** | 0.1 | 1E-12 |
| MeBa079 | Chitinophagales | *Niastella* | 7.0E+05 | 8.1E+05 | **0.3** | 0.4 | 5E-01 |
| **MeBa003** | Actinomycetales | *Pseudarthrobacter* | 3.4E+06 | 2.8E+06 | **0.3** | 0.0 | 4E-09 |
| **MeBa083** | Xanthomonadales | *Lysobacter* | 9.4E+06 | 8.3E+06 | **0.2** | 0.0 | 9E-10 |
| **MeBa054** | Burkholderiales | *Rhizobacter* | 9.4E+06 | 9.0E+06 | **0.1** | 0.0 | 4E-04 |
| MeBa094 | Sphingomonadales | *Sphingomonas* | 3.9E+06 | 3.8E+06 | **0.1** | 0.0 | 8E-02 |
| MeBa028 | Sphingomonadales | *Sphingopyxis* | 6.0E+06 | 5.9E+06 | **0.1** | 0.0 | 2E-01 |
| MaBi325 | Burkholderiales | *Paraburkholderia* | 2.8E+06 | 2.7E+06 | **0.0** | 0.0 | 8E-01 |
| MaBi162 | Burkholderiales | *Nitrosospira* | 1.2E+06 | 1.4E+06 | **-0.1** | 0.0 | 2E-01 |
| MeBa059 | Sphingomonadales | QFOB01 | 2.4E+06 | 2.5E+06 | **-0.1** | 0.0 | 8E-02 |
| **MaBi346** | Sphingomonadales | *Sphingobium* | 3.7E+06 | 3.9E+06 | **-0.1** | 0.0 | 1E-02 |
| **MaBi322** | Sphingomonadales | *Sphingobium* | 3.0E+06 | 3.3E+06 | **-0.2** | 0.0 | 4E-05 |
| **MeBa004** | Sphingomonadales | *Sphingobium* | 3.2E+06 | 3.6E+06 | **-0.2** | 0.0 | 4E-05 |
| **MaBi348** | Sphingomonadales | *Sphingobium* | 2.8E+06 | 3.1E+06 | **-0.2** | 0.0 | 2E-05 |
| **MeBa045** | Sphingomonadales | *Sphingobium* | 1.5E+06 | 1.7E+06 | **-0.2** | 0.0 | 3E-07 |
